# The Bladder as a Target for PCB Toxicity: Evidence from PCB Levels, Phase I Metabolite Levels, and Cytochrome P450 Expression Following Developmental Exposure to a Human-Relevant PCB Mixture in Mice

**DOI:** 10.1021/acs.chemrestox.5c00431

**Published:** 2026-01-22

**Authors:** Hui Wang, Elaine A. Schumacher, Audrey Spiegelhoff, Conner L. Kennedy, Monica M. Ridlon, Rachel F. Marek, Kimberly P. Keil Stietz, Hans-Joachim Lehmler

**Affiliations:** † Department of Occupational and Environmental Health, 50692 The University of Iowa, Iowa City, Iowa 52242, United States; ‡ Department of Comparative Biosciences, 5228 University of Wisconsin-Madison, Madison, Wisconsin 53706, United States; § Department of Civil and Environmental Engineering, 4083 The University of Iowa, Iowa City, Iowa 52242, United States

## Abstract

Lower urinary tract dysfunction is multifactorial, yet the role of environmental exposure remains poorly investigated. Developmental exposure to polychlorinated biphenyls (PCBs) has been linked to altered voiding in mice; however, the disposition of PCBs in the bladder, their bioactivation, and their effects on cytochrome P450 (CYP) expression remain unclear. We exposed mice to an environmentally relevant PCB mixture via maternal diet during gestation and lactation (vehicle, 0.1, 1, or 6 mg/kg/day). Offspring were euthanized at 6 to 7 weeks of age. PCB and hydroxylated PCB (OH-PCB) levels were quantified in the bladder, liver, blood, and urine. CYP expression was measured in the bladder and liver. PCBs and OH-PCBs accumulated in all tissues in dose- and sex-dependent manners, with higher-chlorinated congeners (e.g., PCB118, PCB138, PCB153, and PCB180) preferentially retained. Females exhibited greater hepatic accumulation, reduced urinary elimination, and distinct CYP regulation characterized by increased hepatic and decreased bladder expression. These findings, for the first time, define the signature of PCBs and OH-PCBs in the bladder and reveal a sex-specific PCB disposition and CYP responses. Our results provide new mechanistic insights into developmental PCB exposure and its potential contribution to voiding dysfunction in wildlife, domestic animals, and humans.

## Graphical abstract

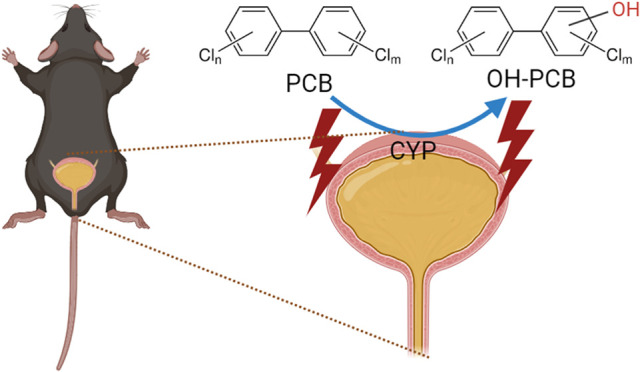

## Introduction

Lower urinary tract symptoms (LUTS) encompass a range of issues related to urine storage, voiding, and postvoiding. Common LUTS include incontinence, detrusor overactivity/underactivity, frequent nighttime urination, weak stream or incomplete emptying, painful urination, and sensations of fullness. In a recent meta-analysis, the global prevalence of LUTS was estimated at 63%, which has increased since the 1990s.^1^ These symptoms significantly impact the quality of life^2,3^ and, in the United States, are attributed to billions of dollars in annual healthcare costs.^4^ While the aging population is predominantly affected by LUTS, they are comorbid with several other health conditions^5^ and are prevalent in children and adults with neurodevelopmental disorders.^6,7^ Issues with bladder storage and voiding extend beyond humans and are also common in companion animals.^8,9^ Little is known about voiding dysfunction in wildlife, where these symptoms may impair fitness, disrupt behaviors, and act as sensitive indicators of environmental contamination that could impact ecosystem health.

The etiology underlying LUTS is complex and remains poorly investigated.^10,11^ Environmental contaminants are hypothesized risk factors for lower urinary tract dysfunction,^12,13^ and animal models have demonstrated that developmental exposures to polychlorinated biphenyls (PCBs) can alter the pathophysiology of the prostate and bladder and can impact voiding physiology.^14–17^ PCBs are a group of man-made organic chemicals that were once widely used in many industrial applications, including electrical equipment, hydraulic systems, plasticizers, paints, sealants, and caulks.^18^ The production of PCBs in the US was banned in 1979 due to concerns about the environmental persistence, bioaccumulation, and toxicity of PCBs. Some PCBs are still produced inadvertently.^19^ As a result, PCBs are still present in the environment and accumulate in foods such as fish and dairy.^20,21^ Population and animal studies have linked PCB exposure to many health concerns, e.g., cancer, endocrine disruption, neurotoxicity, immunosuppression, and developmental and reproductive issues.^18,22,23^ Yet the role of PCBs and their metabolites in urinary health remains largely unexplored.

PCBs are metabolized to hydroxylated metabolites (OH-PCBs) by cytochrome P450 (CYP) enzymes and are known to induce the expression of hepatic P450 enzymes involved in their metabolism.^24^ Although PCBs are assumed to be primarily metabolized by CYPs expressed in the liver, CYPs expressed in the bladder may contribute to the local formation of PCB metabolites.^25^ PCBs are typically eliminated as their metabolites in the feces and, for lower-chlorinated PCBs, in the urine.^26^ Both PCBs and OH-PCBs are developmental neurotoxicants^27^ and have been shown to alter the physiology of many tissues, including the intestine^28^ and vasculature.^29^ Since lower urinary tract function relies on complex interactions between the central and peripheral nervous systems and smooth muscle physiology, it is likely that PCBs and their metabolites could alter bladder function via these pathways as well. We have demonstrated that developmental exposure to the MARBLES mix, an environmentally relevant PCB mixture comprising 12 congeners that mimics the profile of PCBs found in the serum of pregnant women at risk of having a child with autism,^30^ affects voiding physiology in both male and female offspring mice.^14^ Using the same exposure paradigm, we have also demonstrated that PCBs are detectable in the bladders of male offspring at 4 weeks of age following maternal exposure to PCBs.^31^

It is unknown whether PCBs and their metabolites are present in bladder tissue and urine at the time of abnormal voiding, whether their profiles differ by sex or across tissues, and whether, as in the liver, environmental factors like PCBs alter CYP expression in the bladder. To address this, we exposed offspring mice to the MARBLES PCB mixture through their maternal diet during gestation and lactation. PCB levels in the bladder, urine, blood, and liver were quantified using gas chromatography with tandem mass spectrometry (GC-MS/MS), and the expression of CYPs in the bladder and liver was evaluated by quantitative real-time PCR (RT-qPCR). We, for the first time, unveil the metabolic signature of PCBs and OH-PCBs in bladder, urine, blood, and liver of 6-week-old mice following PCB exposure linked to voiding dysfunction and provide evidence that PCB metabolism via phase I enzymes such as CYPs may occur in the bladder and warrant further mechanistic study to link to functional changes in voiding function.

## Materials and Methods

### Chemicals and Analytical Standards

The nomenclature of PCBs in this manuscript follows the naming of PCB congeners according to the U.S. EPA.^32^ For the OH-PCBs, the abbreviations proposed by Maervoet and coauthors were adopted, which use the parent PCB number, followed by the position of the OH group on the biphenyl moiety.^33^ All other chemicals were purchased at the highest grade available from commercial sources. The PCB standards were obtained either from AccuStandard (New Haven, CT), Wellington Laboratories (Guelph, ON, Canada), or synthesized and authenticated in our lab. Detailed lists of chemical and analytical standards are provided in the Supporting Information. The SMILES structures for all PCB congeners and methoxy (MeO)-PCBs are detailed in Tables S1 and S2.

### Animals and Exposure

All animal procedures were conducted in accordance with the NIH Guide for the Care and Use of Laboratory Animals and were approved by the University of Wisconsin–Madison Animal Care and Use Committee (protocol #V006099). The study design, conduct, and reporting of results followed the ARRIVE guidelines.^34^ Wild-type C57BL/6J mice (RRID:IMSR_JAX:000664, Jackson Laboratories, Bar Harbor, ME) were used in this study and housed in clear plastic cages containing corn cob bedding and maintained on a 12 h light and dark cycle at 22 ± 2 °C. Feed (Diet 2019 for breeders and 2020x for maintenance) (Teklad, Indianapolis, IN) and water were available ad libitum.

Oral daily dosing of MARBLES mix to adult female nulliparous mice for 2 weeks before mating and through gestation and lactation was performed as described previously.^30,31,35^ Briefly, female mice (46 ± 5 days old) were dosed at 0.1, 1, or 6 mg of MARBLES PCB/kg body weight/day or vehicle (*n* = 21, 16, 14, and 16 dams for the 0, 0.1, 1, and 6 mg/kg/d groups, respectively). This exposure paradigm leads to detectable PCB levels in the tissues of mouse offspring, comparable to levels found in post-mortem human brains,^36^ and alters behavioral phenotypes, including voiding, at these PCB doses.^14,31,36^ Furthermore, this dosing paradigm did not alter the time to pregnancy in dams, litter size/survival, or body mass of male or female offspring at 6–7 weeks of age.^14^ The MARBLES mix was chosen since it is derived from the MARBLES (Markers of Autism Risk in Babies Learning Early Signs) study and represents a relevant contemporary mixture of PCBs found in pregnant women.^30,37^ Briefly, the MARBLES mix consists of 12 PCBs at specified percentages: PCB28 (48.2%), PCB11 (24.3%), PCB118 (4.9%), PCB101 (4.5%), PCB52 (4.5%), PCB153 (3.1%), PCB180 (2.8%), PCB149 (2.1%), PCB138 (1.7%), PCB84 (1.5%), PCB135 (1.3%), and PCB95 (1.2%).

PCBs for the animal studies were synthesized and authenticated by the Synthesis Core of the University of Iowa Superfund Research Program as previously described.^30,38^ For the preparation of the MARBLES mix, PCBs were dissolved in organic peanut oil (Spectrum Organic Products, LLC, Melville, NY) at a concentration of 20 mg/mL, which was then diluted into organic peanut butter (Trader Joe’s, Monrovia, CA). The vehicle control consisted of peanut oil and peanut butter only, and the samples were administered to mice on weigh boats for oral consumption. Peanut oil and peanut butter are commonly used as inert lipid-based vehicles in rodent studies and have not been shown to alter any end points of interest in our study. Resulting offspring were weaned at postnatal day (P) 21 and group housed with the same sex and dose littermates until they were euthanized at 6–7 weeks of age (mean age 47 ± 4 days old for animals used in this study). To refine and reduce the number of animals, the tissues used here were generated as part of a larger study that characterized voiding function.^14^

Supporting Table S3 contains the body mass for the animals whose tissues were used in this study (male pups, 26, 22, 22, and 23; female pups, 29, 20, 22, and 19 for the 0, 0.1, 1, and 6 mg/kg/d groups, respectively). No changes were observed in body mass at the time of collection, consistent with our earlier work.^14^ Some mice underwent voiding assays (void spot assay, uroflow, and anesthetized cystometry) before being euthanized for tissue collection for PCB analysis.^14^ Urine was collected as free catch urine upon scuffing animals 1 day prior to voiding testing/collection or was collected from a plexiglass plate after mice freely urinated through a grid during uroflow testing.^14^ Urine was frozen in glass vials. Following euthanasia with CO_2_ or isoflurane, blood was collected via cardiac puncture, placed into glass vials containing 80 μL of 7.5% EDTA, and frozen. Bladder and liver tissues were immediately collected following euthanasia, weighed, snap frozen, and stored at −80 °C. If necessary, samples from multiple animals (2–9 animals) were pooled (*n* = 3 pools/exposure group/sex) to generate the necessary amount of tissue for PCB and metabolite quantification, detailed below.

### PCB and OH-PCB Extraction from Tissues

A liquid–liquid extraction (LLE) method was used to extract PCBs and OH-PCBs. The detailed procedures are described in the Supporting Information and were reported elsewhere.^39^ Briefly, about 30 mg of liver (*n* = 4 mice/exposure group/sex) or pooled bladders (35 ± 9 mg, *n* = 24, 3 pools/exposure group/sex) were homogenized in 3 mL 2-propanol. Following the addition of 10 ng of surrogate standards (SS) to all samples, PCBs and OH-PCBs were extracted using a mixture of diethyl ether and hexane. The organic phase was cleaned with 5 mL of 0.1 M phosphoric acid. The concentrated extract was derivatized with diazomethane at 4 °C overnight. Further cleanup was performed using a sulfuric acid and silica gel (1:2, w/w) cartridge. Lastly, the extracts were spiked with the internal standard (IS) and ready for gas chromatography with tandem mass spectrometry (GC-MS/MS) analysis.

Pooled blood samples (830 ± 50 mg, *n* = 24, 3 pools/exposure group/sex) were extracted similarly but with slight modifications, as described in the Supporting Information. Pooled urine samples (450 ± 170 mg, *n* = 24, 3 pools/exposure group/sex) went through an initial sulfatase (type H-2 from *Helix pomatia*, Sigma-Aldrich, St. Louis, MO) deconjugation by converting PCB conjugates to OH-PCBs. The extraction of PCBs and OH-PCBs in urine samples after deconjugation followed the same procedure as for blood extraction.

### GC-MS/MS Analysis

Levels of PCBs and OH-PCBs were quantified by a GC-MS/MS system (Agilent 7890B GC system coupled with an Agilent 7000D Triple Quad). The compound separation was achieved using an SPB-Octyl capillary column (50% *n*-octyl/50% methyl siloxane, 30 m in length, 0.25 mm inner diameter, and 0.25 μm film thickness; Supelco, Bellefonte, PA).^22^ The instrumental setup is provided in the Supporting Information and was described earlier.^40^ The precursor ions, product ions, and collision energies for each analyte are reported in Table S4.

### Quality Assurance/Quality Control (QA/QC) for PCB Extraction

To ensure the accuracy, precision, and repeatability of the extraction, we implemented QA/QC measures, including the analysis of method blank samples, tissues from animals exposed to the vehicle alone, and an ongoing precision recovery (OPR) standard for each batch of extraction. The mass of each analyte was adjusted by the recoveries of the corresponding SS. PCB15 and PCB117 were utilized as SS for PCBs, and 4′–OH-PCB9, 4-OH-PCB91, and 4′–OH-PCB159 (100 ng/mL each in methanol) were employed as SS for OH-PCBs. Deuterium-labeled d-PCB30 (CDN Isotopes, Quebec, Canada) and PCB204 served as IS (volume correctors). Method detection limits (MDL) and limits of detection (LODs) are calculated using the formulas reported in the Supporting Information. All of the QA/QC results, including the SS recoveries, OPR recoveries, MDL, and LOD, are summarized in Tables S5–S7.

### RT-qPCR in the Bladder and Liver

RNA was isolated from frozen bladder and liver tissue (*n* = 5/group/sex) using Cytiva RNAspin isolation kits per manufacturer’s instructions (Fisher Scientific). Briefly, tissues were homogenized with a pestle in a 1.5 mL microcentrifuge tube in lysis buffer supplied in the kit along with the addition of 10 μL of molecular grinding resin (G Biosciences, St Louis, MO). RNA concentrations were determined with the Qubit RNA high sensitivity assay kit, and RNA integrity was assessed with the Qubit RNA IQ assay kit using a Qubit 4 Fluorometer (Invitrogen, Thermo Fisher Scientific). A total of 350 ng of RNA was used in reverse transcription reactions to produce cDNA using the GoScript Reverse Transcription Kit according to the manufacturer’s instructions (Promega, Madison, WI). Real-time PCR was performed using the SsoFast EvaGreen Supermix (Bio-Rad, Hercules, CA). A mixture of 12 μL reactions consisted of 6 μL of SsoFast, 0.5–1 μL of forward and reverse primer combined (from 10 μM primer stock), 3–3.5 μL of water, and 2 μL of cDNA. Primers were designed using Primer3 or NCBI Primer Design, utilizing the NCBI gene mRNA sequence. Primers were entered into Primer BLAST to confirm the target and ordered from IDT (Coralville, IA). Primer efficiency was assessed through serial dilutions of cDNA to determine the optimal annealing temperature. Efficiencies of 90 to 111% were obtained for all primers used. Table S8 lists the primers used along with their corresponding annealing temperatures. Samples were run in triplicate on a CFX Maestro real-time system with Bio-Rad CFX software. The PCR protocol consisted of 95 °C for 2 min, then 39 cycles of 95 °C for 5 s, the annealing temperature specified in Table S8 for 30 s, followed by a final 95 °C for 5 s, 65 °C for 5 s, and 95 °C for 5 s to generate a melt curve. Relative mRNA abundance was determined using the delta CT method as described,^41^ and normalized to *Ppia* (bladder) or *Pgk1* (liver) abundance. Some low-abundance transcripts did not amplify in all samples, or outliers were detected as described in the statistics section. In the final analysis, a total of 3–5 mice per exposure group/sex derived from 3–5 litters/group were used.

### Statistical Analysis

Statistics were performed in GraphPad Prism (Version 10.0.3, RRID:SCR_002798) or R (Version R4.4.2 RRID:SCR_001905). To compare the combined PCB and OH-PCB profiles of two groups, the similarity coefficient cos θ (ranging from 0–1) was calculated using this formula

$$\cos\theta =\frac{\sum_{i=1}^{n}\left(A_{i}B_{i}\right)}{\sqrt{\sum_{i=1}^{n}\left({A_{i}}^{2}\right)}\sqrt{\sum_{i=1}^{n}\left({B_{i}}^{2}\right)}}$$

where A_i_ and B_i_ are the i^th^ components of data set A and B, respectively. For comparing PCB levels in two groups (e.g., male vs female), a two-tailed Student’s *t* test was implemented. For RT-qPCR, Prism’s ROUT method was used to identify and remove outliers. The data were then assessed using a linear mixed effects model using the lme function in R (m = lme (Value ∼ Treatment * Sex, random = ∼1| Litter/Animal, data = data)) and anova function anova­(m). *P* values of main effects of sex, dose, or interaction are reported in the figures. Dunnett’s post hoc comparisons test using the emmeans function in R was only performed on data that had a significant dose or interaction term. Statistical tests were performed on each data set, and *n* values are stated within figure legends. *P* values <0.05 were considered significant and are indicated by asterisks in the figures and reported in the results. The body mass of mice used in this study (Table S3) was assessed via a Kruskal–Wallis test.

## Results and Discussion

### PCB and OH-PCB Levels in the Bladder

Developmental exposure to MARBLES PCB results in sex- and dose-dependent alterations in voiding function in male and female offspring at 6–7 weeks of age.^14^ These changes in voiding are observed without signs of overt toxicity, with no changes in offspring body mass or urine specific gravity (indicative of normal kidney function). To understand the underlying mechanisms driving sex- and dose-dependent effects on voiding function, we here determine whether PCBs or their metabolites are detectable in bladder tissue and urine at this time point (Figures 1 and 2). Because bladder PCB profiles and levels are rarely reported in the literature, blood and liver were investigated in parallel to facilitate a comparison with published PCB and metabolite profiles and levels in humans, companion animals, and wildlife.

**Figure 1:**
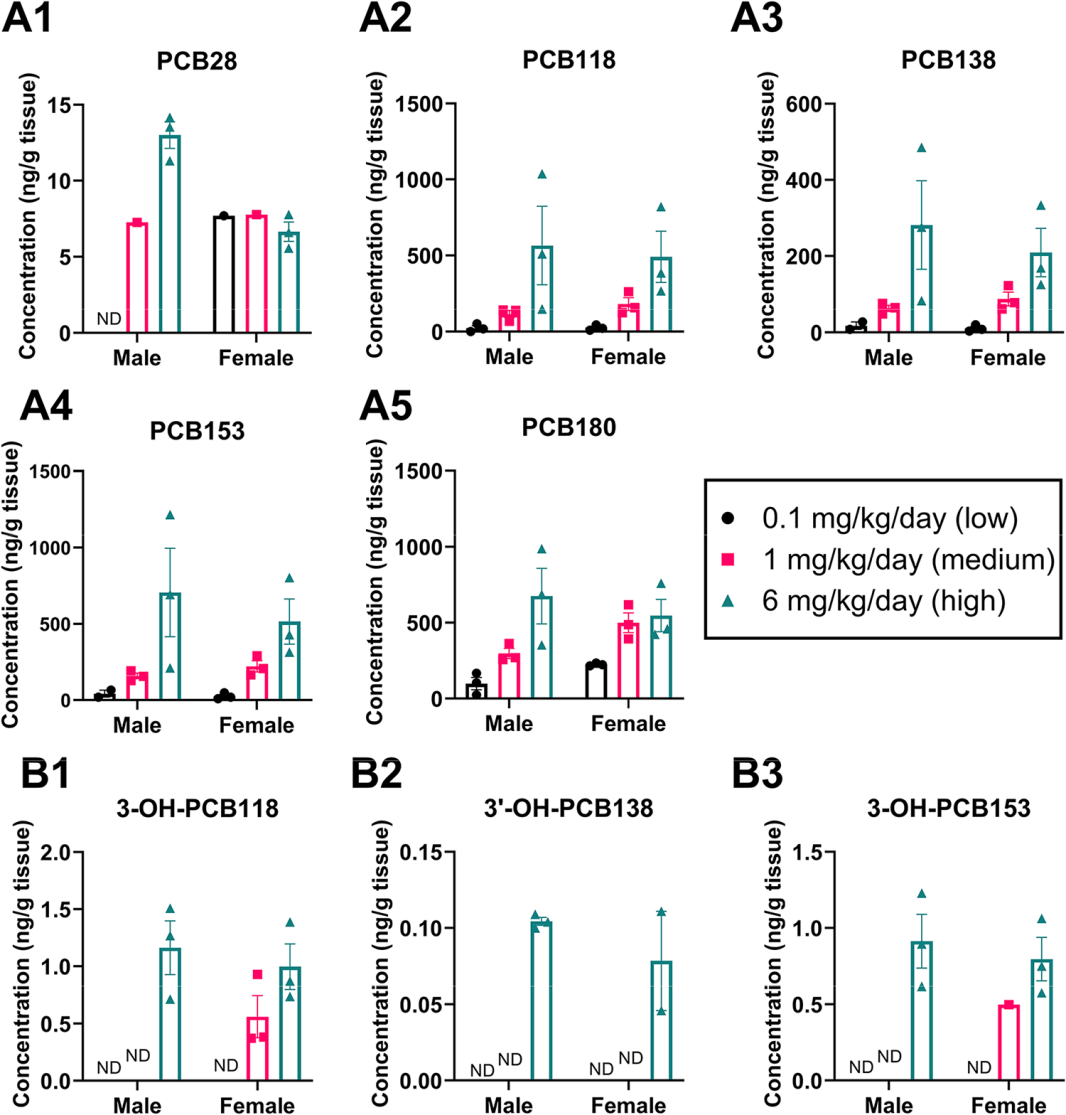
Tissue weight-adjusted concentration (ng/g tissue) of PCBs (A1–A5) and OH-PCBs (B1–B3) detected in pooled offspring bladders. Data are shown as mean ± standard error (*n* = 3 pools/group). Values below LOD are nondetectable (ND) in the figures.

**Figure 2:**
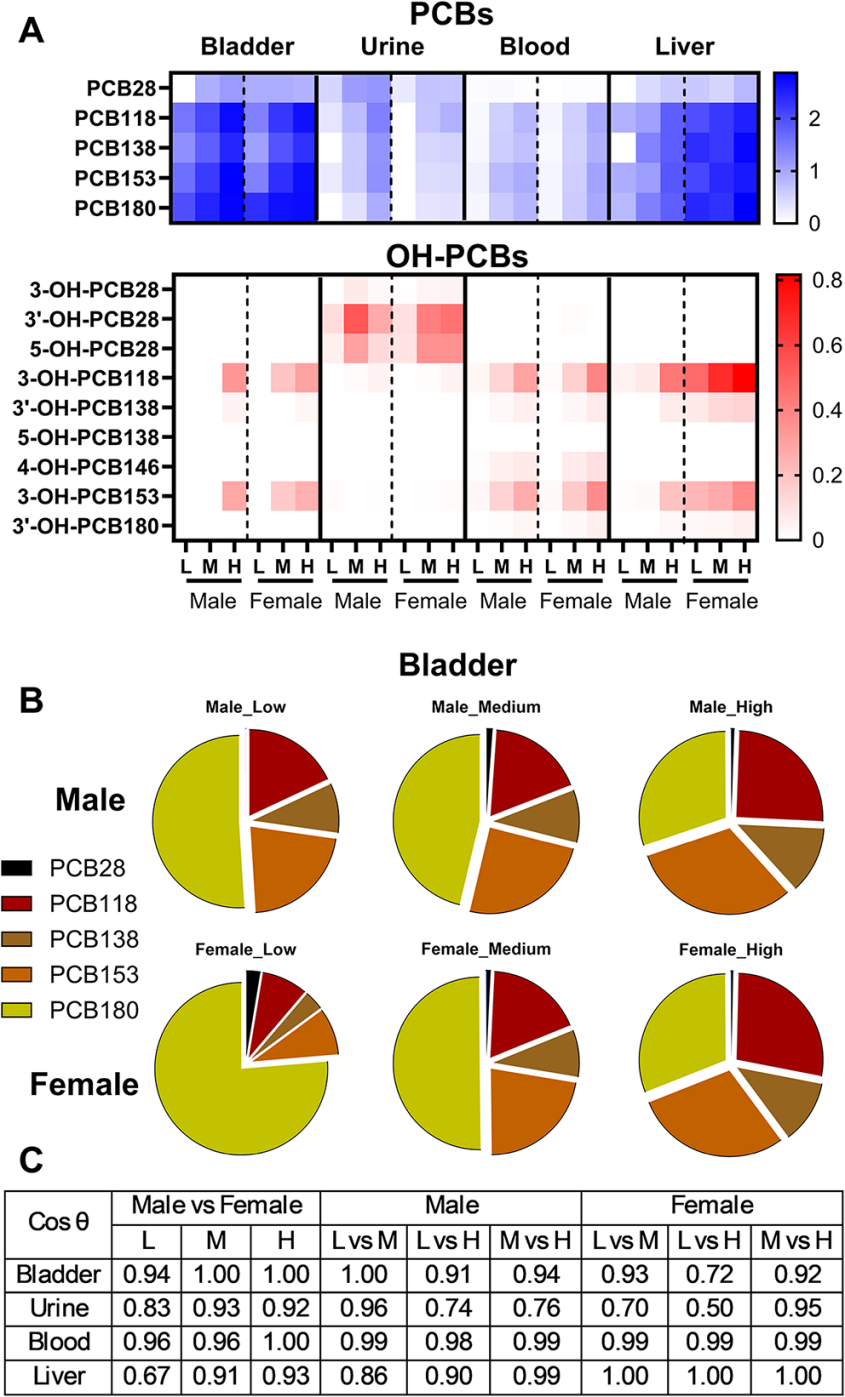
Average levels of PCBs and OH-PCBs (A) in offspring bladder, urine, blood, and liver after developmental exposure to MARBLES mix are illustrated in a heatmap. Values are log-transformed. (B) PCB composition in the bladder is displayed in pie charts for male and female offspring. (C) The similarity coefficient cos θ was calculated between different sexes and dosing groups in each tissue to compare the PCB and OH-PCB composition. L: 0.1 mg/kg/day, M: 1 mg/kg/day, and H: 6 mg/kg/day.

In the bladders, 5 of the 12 parent congeners, PCB28, PCB118, PCB138, PCB153, and PCB180, were detected in both male and female bladders, with higher levels observed in the medium- and high-dose groups (Figures 1A and 2A and Table S9). PCB153 and PCB180 had the highest levels in the bladder, whereas PCB28, a major constituent of the MARBLES mix, displayed the lowest levels in the bladder. The rank order of PCB concentration in male bladder was PCB153 > 180 > 118 > 138 > 28, and in female bladder PCB180 > 153 > 118 > 138 > 28, in the high-dose group. The PCB composition in the bladder, expressed as a mass percentage, changed with an increasing dose (Figure 2B). Briefly, the proportion of PCB180 decreased as the dose increased, whereas the proportion of PCB118 and 153 increased. An earlier study using the same exposure paradigm detected eight out of 12 PCB congeners, including PCB congeners that are more readily metabolized,^39,42^ in the bladder of male offspring at 4 weeks of age.^31^ In the earlier study, PCB levels were ranked as follows: PCB118 > 153 > 28 > 180 > 138 > 52 > 101 > 11. Surprisingly, in our study, the levels of PCB138 (281 vs 235 ng/g), PCB153 (700 vs 607 ng/g), and PCB180 (680 vs 310 ng/g) are higher, while PCB28 (13 vs 600 ng/g) and PCB118 (570 vs 812 ng/g) have lower levels than in the earlier study, in the high dose groups. The differences in PCB levels and their rank order between the two studies are attributed to the age at which the samples were collected.

Four OH-PCBs, including 5,6-OH-PCB11, 3-OH-PCB118, 3-OH-PCB153, and 3′-OH-PCB138, were detected in bladder tissue in a dose-dependent manner in at least one exposure group (Figures 1B and 2A and Table S9). The detection frequency of 3-OH-PCB118 and 3-OH-PCB153 was sex-dependent. Both metabolites were detected only in males from the high-dose group, while they were found in females from both the medium- and high-dose groups. Levels ranged from < LOD to 1.2 ng/g for 3-OH-PCB118 and < LOD to 0.9 ng/g for 3-OH-PCB153. The levels of OH-PCBs in the bladder have not been reported elsewhere. However, earlier studies detected 3-OH-PCB118, 3-OH-PCB153, and 3′-OH-PCB138 in the urine or serum of humans^40,43^ and animals.^43^ Earlier mouse studies using a similar dosing paradigm for 7 weeks have reported the presence of 3-OH-PCB118, 3-OH-PCB153, and 3′-OH-PCB138 in the tissues of female mice.^44^

The similarity coefficient, cos θ, was used to explore dose- and sex-dependent differences in the PCB and OH-PCB profiles of the bladder (Figure 2C). Female mice exposed to the low dose exhibited a distinct profile compared with the higher PCB dose groups. Specifically, the cos θ values were 0.93 for the low dose versus the medium dose, and 0.72 for the low dose compared to the high dose (Figure 2C). In contrast, the PCB and OH-PCB profiles in male bladders were more comparable across exposure groups. Additionally, profiles in the female bladder differed from those in the male bladder in the low-dose group (cos θ = 0.94), indicating sex-dependent differences in how PCBs are distributed in the mouse bladder, at least at lower doses. Overall, our results demonstrate that developmental exposure to PCBs via the maternal diet results in detectable levels of PCBs and OH-PCBs in the bladders of male and female mice at 6–7 weeks of age, with distinct dose and sex differences.

### PCB and OH-PCB Levels in the Urine

Urine PCB levels were determined because urine is a route of elimination for PCB metabolites, especially for lower-chlorinated PCBs,^26,45^ and to confirm whether bladder tissue profiles simply reflect urine profiles. In the urine, 8 of the 12 parent congeners, including PCB28, PCB95, PCB101, PCB118, PCB135, PCB138, PCB153, and PCB180, were detected at levels above the limit of detection in at least one exposure group (Figures 2A and S1A and Table S10). In males with high PCB exposure, the concentration rank order of PCBs in urine was PCB118 > 153 > 138 > 28 > 180 > 101. In females, the order was PCB118 > 28 > 138 > 153 > 180. In urine samples, PCB95 and PCB135 were detected only in females exposed to the medium PCB dose, whereas PCB101 was uniquely identified in the male group exposed to the high PCB dose (Table S10). Parent PCBs have been previously detected in urine;^45^ however, many of these studies utilized bile duct cannulated animals or metabolic cages^45,46^ and, unlike our study, did not collect urine directly. This raises the possibility that the PCBs detected in those studies resulted from contact between urine and fecal material, complicating the interpretation of their findings.

Several OH-PCB metabolites of PCB28, including 2′-OH-PCB28, 3-OH-PCB28, 3′-OH-PCB28, and 5-OH-PCB28, as well as 3-OH-PCB118, 3-OH-PCB153, and 3′-OH-PCB138, were detected in the urine after deconjugation with type H-2 sulfatase from *H. pomatia* in at least one exposure group (Figure S1B and Table S10). These results are consistent with the established structure–disposition relationship, suggesting that PCB congeners with fewer chlorine atoms are primarily eliminated as PCB conjugates via the urine, whereas those with a higher number of chlorine atoms are more likely to be excreted in feces.^26^ The three OH-PCB28 metabolites account for most of the total OH-PCBs in urine (Figure 2A). In a study sampling human urine, 3-OH-PCB28 was detected as the most predominant congener, suggesting urinary OH-PCBs are a potential biomarker of exposure to PCB28,^43^ analogous to studies of inhaled PCB3 in rats.^46^ Furthermore, in a previous study that exposed adult female mice to MARBLES mix for 7 weeks, 3-OH-PCB28, 3′-OH-PCB28, and 5-OH-PCB28 were detected in the feces, indicating fecal elimination of PCB28 metabolites as an additional route for PCB28 clearance.^47^

OH-PCB levels also revealed some sex differences in the urine. Briefly, the 2′-OH-PCB28 metabolite was detected in only female mice from the medium- and high-dose groups. The 3′-OH-PCB138 was found in the urine of male mice across all dose groups, but it was not present in female urine (Table S10). In the urine of male mice, levels of 3-OH-PCB28, 3′-OH-PCB28, and 5-OH-PCB28 after deconjugation were highest in the medium-exposure group compared to both the low- and high-exposure groups. Because urinary OH-PCBs were measured after deconjugation, additional studies are needed to determine whether the sex- and dose-dependent differences in OH-PCB levels reflect differences in the excretion of the free OH-PCBs or their conjugates. Using the similarity coefficient, PCB and OH-PCB urine profiles displayed dose-dependent effects. Female mice exposed to low dose exhibited a distinct profile compared to medium and high PCB dose groups (cos θ values 0.70 and 0.50, respectively) (Figure 2C). Male mice exposed to the high dose exhibited a distinct profile compared to low and medium PCB dose groups (cos θ values 0.74 and 0.76, respectively) (Figure 2C). Sex differences were most pronounced in the low-exposure group (cos θ = 0.83, Figure 2C).

### PCB and OH-PCB Levels in the Blood and Liver

For comparison purposes, PCBs and metabolites were also quantified in the blood and livers of mice developmentally exposed to the MARBLES mix. Whole blood PCB concentrations likely underestimate serum PCB levels, which are frequently reported by human biomonitoring studies, on a volume- or weight-normalized basis because PCBs preferentially partition into serum lipids.^48^ However, whole blood provides an integrated measure of the circulating PCB burden and allows valid comparisons across exposure groups within the study. In the blood, 7 of the 12 parent congeners, including PCB28, PCB101, PCB118, PCB138, PCB149, PCB153, and PCB180, were detected in at least one exposure group (Figure S2A and Table S11). In the high-dose group, the rank order of PCB concentrations in blood from male mice was PCB153 > 180 > 118 > 138 > 28. In female blood, the rank order was PCB153 > 180 > 118 > 138 > 101 > 28 > 149 (Figure S2A and Table S11). Several OH-PCBs, including 4-OH-PCB11, 3′-OH-PCB28, 3-OH-PCB118, 3-OH-PCB153, 4-OH-PCB146, 3′-OH-PCB138, 5-OH-PCB138, and 3′-OH-PCB180, were detected in the blood of at least one exposure group (Figure S2B and Table S11). Only two OH-PCBs showed sex differences in their detection frequency. 4-OH-PCB11 was detected in blood from female mice from the high PCB dose, and 3′-OH-PCB28 was observed only in the female mice exposed to the medium dose. Using the similarity coefficient, PCB and OH-PCB profiles showed no dose-dependent differences but displayed small sex differences in the low- and medium-exposure groups (cos θ = 0.96 and 0.96, respectively, Figure 2C).

In the liver, 5 of the 12 parent congeners, including PCB28, PCB118, PCB138, PCB153, and PCB180, were detected in both the male and female livers (Figure S3A and Table S12). In male mice exposed to the high PCB dose, the rank order of PCB concentration in the liver was PCB153 > PCB180 > PCB118 ≈ PCB138 > PCB28. In female mice, a different rank order was observed, with PCB180 > PCB138 > PCB153 > PCB118 > PCB28 (Table S12). These sex differences in the rank order reflect statistically significant differences in PCB118, PCB138, and PCB180 levels between male vs female mouse livers (*p* < 0.01, Student’s *t* test), ranging from 1.9-fold for PCB118 to 3.6-fold for PCB138 (Figure S4). Additionally, 3-OH-PCB118, 3-OH-PCB153, 3′-OH-PCB138, and 3′-FooOH-PCB180 were detected in at least two exposure groups (Figure S3B and Table S12). Overall, PCB and OH-PCB profiles displayed sex differences, particularly in the low exposure group (cos θ = 0.67) (Figure 2C). Small dose-dependent differences in the profiles were observed in the male liver from mice exposed to the low and medium doses of the MARBLES mix (cos θ = 0.86 and 0.90, respectively), whereas profiles in female mice were identical irrespective of the exposure group (Figure 2C).

### Comparison of PCB and OH-PCB across Compartments

A comparison of PCB levels across the compartments investigated reveals that particularly high-chlorinated parent PCBs, such as PCB180, PCB153, PCB118, and PCB138, exhibit elevated levels in the bladder and liver compared with blood and urine (Figures 2A and S4). These findings are consistent with earlier studies that demonstrate a direct relationship between PCB tissue levels and tissue fat content.^49^ Interestingly, although PCB28 is the most abundant congener (48.2%) in the MARBLES mix, it showed minimal deposition in the bladder, blood, or liver compared to higher-chlorinated congeners mentioned above. Urine, in contrast, has a higher proportion of PCB28. Consistent with the rapid metabolism of PCB11 in rodents,^42^ PCB11 was not detected in any tissues or other biological matrices, despite accounting for almost one-quarter (24.3%) of the MARBLES PCB mix. Similarly, PCB52 and PCB84, two PCB congeners that are readily metabolized in rodents,^39,50^ were also not detected in any of the examined compartments. A comparison of the OH-PCB levels across the compartments investigated showed the retention of some OH-PCBs in blood, with eight OH-PCBs detected compared to only four OH-PCBs each in the bladder and liver (Table S9, S11 and S12).^51^

### Expression of Drug-Metabolizing Enzymes in the Bladder

Since we observed differences in the parent PCBs or their metabolites present in the bladder, we tested whether PCB exposure altered the expression of nine CYP genes and one UDP-glucuronosyltransferase (*Ugt2b1*) potentially involved in PCB metabolism in the bladder (Figure 3) or liver (Figure 4) of 6-week-old mice developmentally exposed to the MARBLES mix.

**Figure 3:**
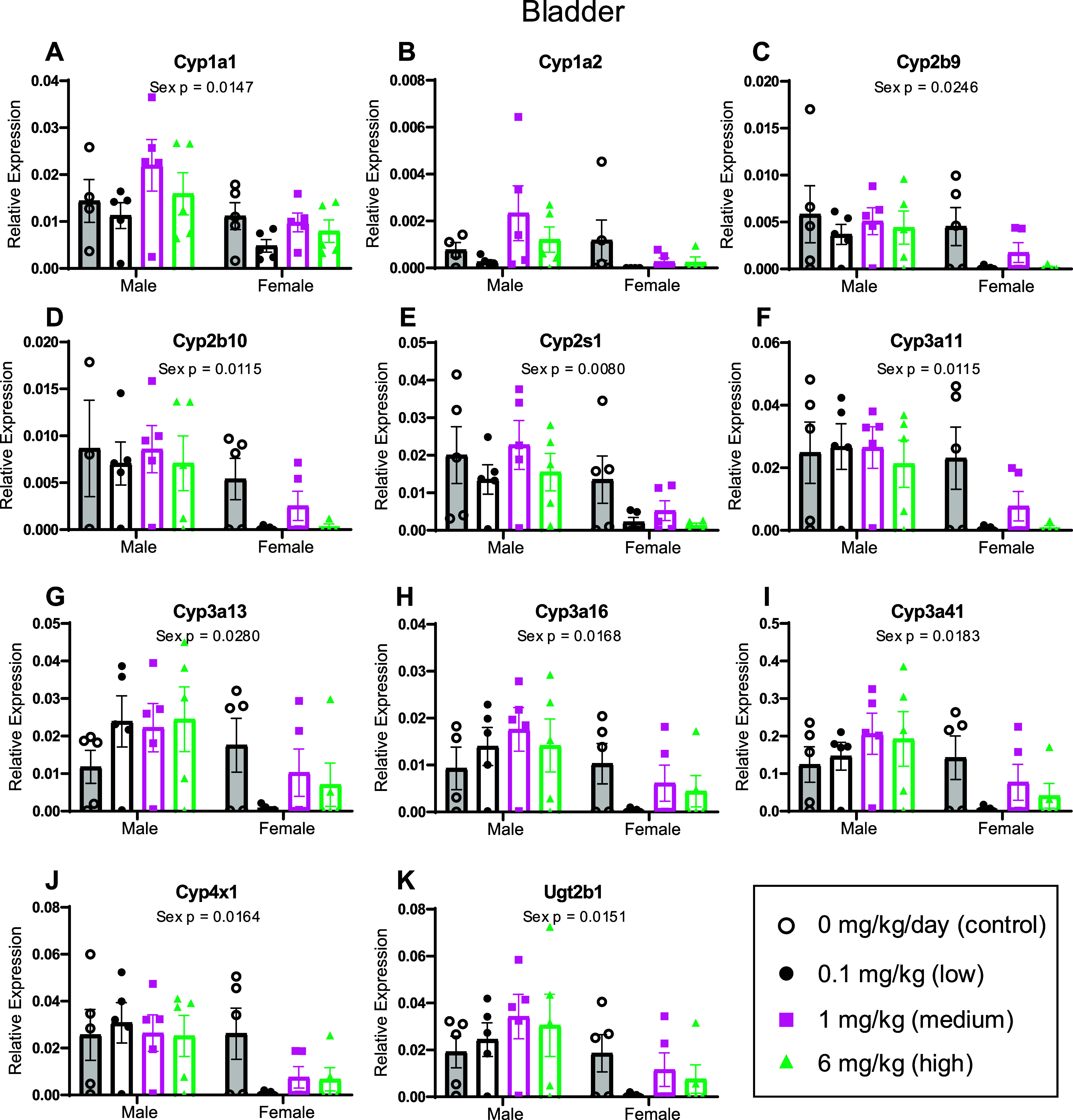
Relative expression of *Cyp* and *Ugt* genes in offspring bladder using RT-qPCR. Following developmental exposure to the MARBLES mix, bladders were collected from offspring at 6 weeks of age to assess the mRNA abundance of *Cyp* and *Ugt* genes. Data are presented as mean ± SEM, *n* = 3–5 mice per group. Data were assessed using a linear mixed effects model and anova function in R. Prism’s ROUT method was used to identify and remove outliers. *P* values of main effects are reported in each graph; *p*-values <0.05 were considered significant.

**Figure 4:**
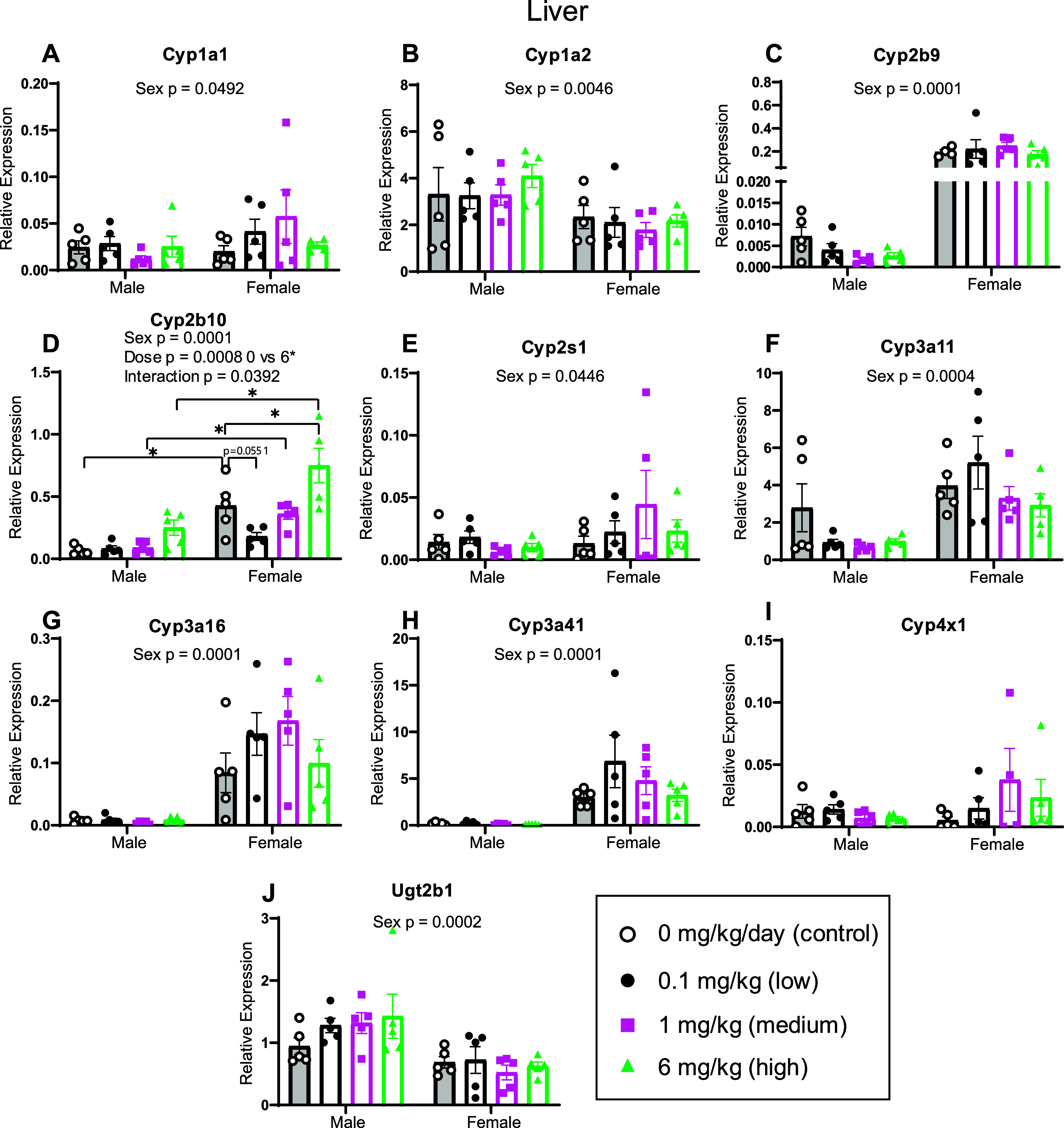
Relative expression of *Cyp* and *Ugt* genes in offspring liver using RT-qPCR. Following developmental exposure to the MARBLES mix, livers were collected from offspring at 6 weeks of age to assess the mRNA abundance of *Cyp* and *Ugt* genes. Data are presented as mean ± SEM, *n* = 4–5 mice per group. Data were assessed using a linear mixed effects model and anova function in R followed by Dunnett’s multiple comparisons test when dose or interaction factors were significant. Prism’s ROUT method was used to identify and remove outliers. *P* values of main effects are reported in each graph; *p*-values <0.05 were considered significant. Brackets and asterisks indicate significant differences between groups for which a main effect of dose or interaction was present.

In the bladder, there was a significant main effect of sex on the expression of *Cyp1a1*, *Cyp2b9*, *Cyp2b10*, *Cyp2s1, Cyp3a11*, *Cyp3a13*, *Cyp3a16*, *Cyp3a41*, *Cyp4 × 1*, and *Ugt2b1* (Figure 3). There were no significant effects on the expression of *Cyp1a2* in the bladder. For all genes with significant sex effects, female expression was lower than that of male expression. Although there were no significant interaction terms and no post hoc analyses were conducted, the sex effects of decreased expression of drug-metabolizing genes in female versus male bladders appeared to be largely driven by the PCB exposure groups. Together, these data indicate that CYP expression varies by sex in the bladder but is not altered by PCBs alone. Sex differences in CYP expression have been reported previously in the lower urinary tract. For example, higher basal levels of *Cyp2e1* are observed in the male mouse kidney compared to the female mouse kidney.^52^ While we did not examine *Cyp2e1* here, these earlier findings, together with our findings, suggest that bladder-specific expression of CYP enzymes may influence xenobiotic metabolism and help explain some of the sex effects observed upon exposure to various lower urinary tract toxicants.

It is unknown whether environmental factors, such as PCBs, affect the expression of CYPs and other drug-metabolizing enzymes in the bladder or if local metabolism contributes to bladder toxicity. CYPs are known to be present in the urothelium of healthy bladder and in bladder cancer biopsies.^25^ CYP function has been linked to the differentiation status of the urothelium.^53^ During the process of urothelial cell differentiation, expression of NADPH P450 oxidoreductase (POR), the electron donor for CYPs, is associated with increased activity of CYP1A1 and CYP1B1 in human urothelial cell lines.^53^ Interestingly, in muscle-invasive bladder cancers, Cyp1 activity was low in bladder cancers with low POR abundance and higher in luminal cells with higher POR activity.^53^ We previously reported that developmental exposure to the MARBLES mix did not induce bladder cell death or change the cellular composition or expected populations of cell types present in the urothelium in offspring at 4 weeks of age.^31^ Thus, we do not expect that the differentiation status of the urothelium was altered in this study. However, we did not assess whether PCBs directly influence POR activity or whether this, in turn, influences CYP activity.

### Hepatic Expression of Drug-Metabolizing Enzymes in the Liver

There was a significant main effect of sex in the expression of *Cyp1a1, Cyp1a2*, *Cyp2b9*, *Cyp2b10*, *Cyp2s1, Cyp3a11*, *Cyp3a16*, *Cyp3a41*, and *Ugt2b1* (Figure 4). There were no significant effects on the expression of *Cyp4 × 1*. For the genes with significant sex effects, female expression was decreased compared to male expression for *Cyp1a2* and *Ugt2b1* (Figure 4B,J). In contrast, female expression was increased compared to that of males for *Cyp1a1, Cyp2b9*, *Cyp2b10*, *Cyp2s1, Cyp3a11*, *Cyp3a16*, and *Cyp3a41* (Figure 4C,D, F–H). In addition to a sex effect, *Cyp2b10* also exhibited a significant main effect of dose, with the high-dose group showing a significant increase in expression compared to the vehicle control (Figure 4D). There was also a significant interaction between the dose and sex for liver *Cyp2b10* expression. Post hoc analysis revealed significantly higher expression of *Cyp2b10* in the female liver compared to the male liver in the control, medium-dose and high-dose groups. Moreover, in the female liver, a nearly significant decrease in *Cyp2b10* expression was observed in the low-dose group compared with the vehicle control. Conversely, there was a significant increase in expression in the high PCB dose group compared to the vehicle control. These data indicate that hepatic CYP expression in offspring developmentally exposed to the MARBLES mix depends on sex and PCB dose.

It is well-known that high doses of individual PCB congeners or PCB mixtures alter the expression of hepatic drug-metabolizing enzymes in rodents.^54–56^ The CYP isoforms induced following PCB exposure depend on the structures of the PCB congeners. Briefly, dioxin-like PCB congeners induce CYP1 enzymes, whereas nondioxin-like PCB congeners with multiple ortho chlorines induce CYP2 isoforms. As a result, different CYP isoforms may be induced following exposure to PCB mixtures. For example, the Fox River Mixture of PCBs administered to adult female mice at 6 or 30 mg/kg orally for 3 days induces the expression of hepatic *Cyp1a1*, *Cyp1a2*, *Cyp2b10*, *Cyp2c50*, *Cyp3a16*, and *Cyp3a41a*, which are involved in xenobiotic metabolism.^57^ In contrast to the earlier study, we observed a statistically significant increase in *Cyp2b10* expression only in the high-dose group compared to that in the vehicle control. Several differences may explain these findings. First, we examined the CYP expression in adult offspring that were exposed developmentally, whereas Lim et al.^57^ examined CYP expression in adult female liver immediately after 3 days of dosing. Thus, the timing, duration, and dose of exposure are different in the two studies. Second, we examined the MARBLES mix, which is primarily composed of nondioxin-like PCBs (except PCB118). In contrast, the Fox River Mixture contains a greater number of dioxin-like PCBs, which activate the aryl hydrocarbon receptor (AhR). Therefore, the fact that the Fox River Mixture induced liver expression of Ahr target genes Cyp1a1 and Cyp1a2 in adult female mice, whereas developmental exposure to the MARBLES mix did not, is not surprising.

### Environmental Relevance

PCBs are ubiquitous and persistent environmental pollutants. Developmental exposure to a human-relevant MARBLES mix resulted in sex- and dose-dependent accumulation of parent and hydroxylated PCBs across the bladder, urine, blood, and liver tissues in juvenile mice. Total PCBs levels in juvenile blood (1.5 to 24 ng/g in male and 1.3 to 35 ng/g in female pups) or serum (2.7 to 42 ng/mL for male and 2.3 to 62 ng/mL for female), estimated based on a plasma-to-whole blood distribution coefficient of 1.69 ^58^ and blood density of 1.057 g/mL,^59^ are within the range of total PCB serum levels in pregnant women in the MARBLES cohort (0.7 to 12.6 ng/mL).^30^ Higher-chlorinated congeners were preferentially retained in all tissues and biological matrices, while blood and urine contained more hydroxylated metabolites, reflecting the ongoing biotransformation of PCBs to more water-soluble metabolites. Notably, sex differences were evident in PCB profiles with female mice often showing greater retention and distinct metabolite patterns. Expression of CYP enzymes varied by sex and tissue, with the female liver showing both up- and downregulation of specific CYP genes in a dose-dependent manner. In the bladder, CYP expression was consistently lower in females, suggesting a sex-specific regulation of xenobiotic metabolism.

These findings highlight the bladder as a previously under-recognized metabolic target of developmental PCB exposure and underscore the importance of considering sex as a biological variable in toxicological assessments. Furthermore, these complex alterations in PCB disposition could explain the diverse sex- and dose-dependent effects observed in the lower urinary tracts of offspring mice following developmental MARBLES exposure. Previous studies show that the same developmental MARBLES exposure paradigm used here not only leads to an increase in small voids, an increase in void frequency, and an increase in void pressures in a dose- and sex-dependent manner^14^ but also is capable of increasing nerve fiber density in bladder suburothelium (males, high dose) and increasing the number of CD45+ immune cells and macrophages in offspring bladders (predominant in females at low dose), without signs of overt toxicity.^31,60^ However, this study is limited by its focus on a subset of hydroxylated metabolites, the use of mRNA as a surrogate for enzymatic activity, and the absence of assessments directly linking metabolic changes to voiding. While sex- and dose-dependent voiding dysfunction has been observed in developmentally exposed offspring,^14^ future studies integrating functional assays and broader metabolite profiling are needed to fully understand the impact of developmental PCB exposure on bladder health, including LUTS, in wildlife, domesticated animals, and humans.

## Supplementary Material



## Data Availability

Research data supporting the findings of this study are openly available in Iowa Research Online at 10.25820/data.008178.

## Notes

Additional materials and methods, including PCB extraction and instrumental setup, tables with unique chemical identifiers, animal body mass, instrumental parameters, QA/QC results, primer information, and tables and figures with PCB and OH-PCB levels (PDF)
