# Supplementary material for: The Bladder as a Target for PCB Toxicity: Evidence from PCB Levels, Phase I Metabolite Levels, and Cytochrome P450 Expression Following Developmental Exposure to a Human-Relevant PCB Mixture in Mice
Source: Chem Res Toxicol. 2026 Jan 22;39(2):236–47. doi: 10.1021/acs.chemrestox.5c00431 (PMC12914705; doi:10.1021/acs.chemrestox.5c00431)
Supplement: Supplementary file 1 [file tx5c00431_si_001.pdf]

The bladder as a target for PCB toxicity: Evidence from PCB levels, phase Ⅰ metabolite levels, and cytochrome P450 expression following developmental exposure to a human-relevant PCB mixture in mice

Hui Wang[1], Elaine A. Schumacher[2], Audrey Spiegelhoff[2], Conner L. Kennedy[2], Monica M. Ridlon[2], Rachel F. Marek[3], Kimberly P. Keil Stietz[2], Hans-Joachim Lehmler[1*]*

[1]Department of Occupational and Environmental Health, The University of Iowa, Iowa City, IA 52242, USA; [2]Department of Comparative Biosciences, University of Wisconsin-Madison, Madison, WI 53706, USA; [3]Department of Civil and Environmental Engineering, The University of Iowa, Iowa City, IA 52242, USA

***Co-Corresponding Authorship:**

Dr. Hans-Joachim Lehmler
The University of Iowa
Department of Occupational and Environmental Health
University of Iowa Research Park, #221 IREH
Iowa City, IA 52242-5000, United States of America
Phone: (319) 335-4310
Fax: (319) 335-4290
e-mail: hans-joachim-lehmler@uiowa.edu

Dr. Kimberly Keil Stietz
University of Wisconsin-Madison
Department of Comparative Biosciences
2015 Linden Drive
Madison, WI 53706, United States of America
Phone: (608) 265-2879
Email: kkeil@wisc.edu

Conflicts of interests: The authors declare no competing financial interests.

Table of Contents

Chemicals and analytical standards	S3
PCB and OH-PCB extraction from the offspring liver, bladder, blood, and urine.	S4
GC-MS/MS analysis	S5
Methods to calculate the method detection limits (MDL) and limits of detection (LODs)	S5
**Table S1.** The canonical SMILES structure of all 209 PCB congeners. The molecular structure and IUPAC names were also listed for comparison	S7
**Table S2.** The SMILES structure of 124 available methoxyl-PCBs used in calibration standards as derivative forms for corresponding OH-PCBs.	S14
**Table S3**. The body mass of animals used in this study.	S18
**Table S4.** Precursor ions, product ions, and collision energies for each analyte in the GC-MS/MS analysis.	S19
**Table S5.** Recoveries (%) of surrogate standards for PCB and OH-PCB in different biological matrices.	S21
**Table S6.** Ongoing Precision Recovery (OPR) for selected PCBs and OH-PCBs in method blanks and tissue matrices.	S22
**Table S7.** Method Detection Limits (MDLs) and Limits of Detection (LODs) for PCBs from MARBLES mixture and their possible OH-PCB metabolites.	S23
**Table S8**. RT-qPCR Primer information.	S25
**Table S9**. PCB and OH-PCB levels in offspring bladder (ng/g, n=3).	S26
**Table S10.** PCB and OH-PCB levels in offspring urine (ng/g, n=3).	S27
**Table S11**. PCB and OH-PCB levels in offspring blood (ng/g, n=3).	S28
**Table S12**. PCB and OH-PCB levels in the offspring liver (ng/g, n=4).	S29
**Figure S1.** Weight adjusted concentrations (ng/g) of PCBs (A1-A5) and OH-PCBs (B1-B6) detected in pooled offspring urine.	S30
**Figure S2.** Weight adjusted concentrations (ng/g) of PCBs (A1-A5) and OH-PCBs (B1-B6) detected in pooled offspring whole blood.	S31
**Figure S3.** Weight adjusted concentrations (ng/g) of PCBs (A1-A5) and OH-PCBs (B1-B4) detected in offspring liver.	S32
**Figure S4.** Mass percentage (%) of PCB and OH-PCB detected in high dose (6 mg/kg) PCB-exposed offspring bladder, blood, urine, and liver samples.	S33
References	S34

Chemicals and analytical standards

All solvents (pesticide grade), sulfuric acid (concentrated), hydrochloric acid (concentrated), potassium chloride, sodium chloride, silica gel, and sodium sulfite were purchased from Fisher Scientific (Fair Lawn, NJ, USA). Tetrabutylammonium hydrogen sulfate was obtained from J.T. Baker (Phillipsburg, NJ, USA). A solution of diazomethane in diethyl ether was synthesized using an Aldrich Mini Diazald® Apparatus as described.[1] Individual PCB and OH-PCB standards were purchased from AccuStandard (New Haven, CT, USA) unless otherwise stated. 2,5-Dichlorobiphenyl-4'-ol (4'-OH-PCB9), 3,3'-dichlorobiphenyl-4-ol (4-OH-PCB11), 2,3',4-trichlorobiphenyl-4'-ol (4'-OH-PCB25), 2,2',5,5'-tetrachlorobiphenyl-4-ol (4-OH-PCB52), 2,2',3,4',6-pentachlorobiphenyl-4-ol (4-OH-PCB91), 2,2',3,5',6-pentachlorobiphenyl-4-ol (4-OH-PCB95), and 2,2',3,5',6-pentachlorobiphenyl-5-ol (5-OH-PCB95) were prepared as described.[2-6] 4,4'-Dichlorobiphenyl (PCB15) and 2,3,4',5,6-pentachlorobiphenyl (PCB117) (100 ng/mL each in isooctane) were used as surrogate standards for PCBs, and 4'-OH-PCB9, 4-OH-PCB91, and 2,3,3',4,5,5'-hexachlorobiphenyl-4'-ol (4'-OH-PCB159) (100 ng/mL each in methanol) were used as surrogate standards for OH-PCBs. Deuterium-labeled 2,4,6-trichlorobiphenyl-2',3',4',5',6'-d_5 (d-PCB30; CDN Isotopes, Quebec, Canada) and 2,2',3,4,4',5,6,6'-octachlorobiphenyl (PCB204) were used as internal standards (volume correctors).

Several calibration standards were prepared for peak verification and mass calibration. A PCB calibration standard was prepared from standard mixtures containing all 209 PCB congeners (AccuStandard). Two MeO-PCB calibration standard solutions were used for the analysis of MeO-PCBs. MeO-PCB standard solution 1 contained seventy-two methoxylated PCBs (MeO-PCBs) (70 mono-MeO-PCBs and two di-MeO-PCBs) provided by AccuStandard or Wellington Laboratories (Guelph, ON, Canada). MeO-PCB standard solution 2 consisted of fifty-two MeO-PCBs (28 mono-MeO-PCBs and 24 di-MeO-PCBs as standard Solution 2). These MeO-PCBs

were synthesized with Suzuki coupling reaction between a suitable benzene boronic acid and a methoxylated bromochlorobenzene[6, 7] and authenticated as described.[4, 8-12] In addition, an OH-PCB standard solution was prepared containing 4-OH-PCB11, 4'-OH-PCB25, 4-OH-PCB52, 4-OH-PCB95, 5-OH-PCB95, 2,2',4,4',5,5'-hexachlorobiphenyl-3-ol (3-OH-PCB153), and 2,2',3,4',5,5'-hexachlorobiphenyl-4-ol (4-OH-PCB146) in methanol (100 ng/mL each).

PCB and OH-PCB extraction from the offspring liver, bladder, blood, and urine

A liquid-liquid extraction method was used to simultaneously extract and analyze PCBs and OH-PCBs. Briefly, about 30 mg of liver (n=4 mice/exposure group/sex) or pooled bladders (35±9 mg, n=24, 3 pools/exposure group/sex) were homogenized with 3 mL 2-propanol using a TissueRuptor (QIAGEN, Hilden, Germany). After spiking 10 ng PCB15 and PCB117 and 10 ng OH-PCB (4'-OH-PCB9, 4-OH-PCB91, and 4'-OH-PCB159) as surrogate standards to all samples, PCBs and OH-PCBs were extracted with diethyl ether and hexane (1:9, v/v), followed by washing the organic extracts with 5 mL of 0.1 M phosphoric acid in 0.9% sodium chloride solution. The extracts were concentrated under a gentle stream of nitrogen and derivatized with diazomethane at 4°C overnight.[13] The extracts were passed through a sulfuric acid and silica gel (1:2, w/w) cartridge for further cleanup. Finally, the extracts were concentrated under gentle nitrogen flow and spiked with the internal standard (d-PCB30 and PCB204) for gas chromatography with tandem mass spectrometry (GC-MS/MS) analysis.

Pooled blood samples (830±50 mg, n=24, 3 pools/exposure group/sex) were extracted similarly, but with slight modifications. Briefly, 1 mL of 6 M HCl was added to the serum. After spiking the surrogate standard, PCB and OH-PCB were extracted using a 2-propanol and hexane: methyl tert-butyl ether (MTBE) (1:1, v/v) mixture. After washing with 3 mL 1% KCl, the extracts were concentrated and derivatized as described above. After further cleanup using 2-propanol and

tetrabutylammonium hydrogen sulfate (TBA), the extracts were subjected to the same cleanup steps as described for the extraction of bladders.

Twenty µL of sulfatase (type H-2 from *Helix pomatia*, Sigma-Aldrich, St. Louis, MO, USA) was added to pooled urine samples (450±170 mg, n=24, 3 pools/exposure group/sex) to convert PCB conjugates to OH-PCBs in a shaking water bath for 16 h at 37 °C.[14] The extraction of PCBs and OH-PCBs in urine samples after deconjugation followed the same procedure as described above for blood extraction.

GC-MS/MS analysis

PCBs and OH-PCBs were quantified by a GC-MS/MS system (Agilent 7890B GC system, Agilent 7000D Triple Quad, Agilent 7693 autosampler) equipped with an SPB-Octyl capillary column (50% n-octyl/50% methyl siloxane, 30 m length, 0.25 mm inner diameter, 0.25 µm film thickness; Supelco, Bellefonte, PA).[15] Helium was used as the carrier gas (0.8 mL/min), and nitrogen was used as the collision gas. The gas chromatograph was operated in solvent vent injection mode with the following setup: initial temperature, 45 °C; initial time, 0.06 min; ramp, 600 °C/min to inlet temperature of 325 °C at 5 psi. The oven temperature program of the gas chromatograph was 45 °C for 2 min, 45 to 75 °C at 100 °C/min, hold for 5 min, 75 to 150 °C at 15 °C/min, hold for 1 min, 150 to 280 at 2.5 °C/min, and hold 5 min. The triple quadrupole electron ionization source was set to 230 °C. Congener mass was quantified by applying a relative response factor obtained from the calibration standard for each congener.[16]

Methods to calculate the method detection limits (MDL) and limits of detection (LODs)

Method detection limits (MDL) and limits of detection (LODs) were calculated using the formula reported in the Supporting Information:

$$MDL = mean_{blank} + t_{0.01,\, n-1} * SD_{blank}$$

where $mean_{blank}$ is the mean of method blanks, $t_{0.01,\ n-1}$ is Student's t-value for n – 1 degrees of freedom at the 99% confidence level, and SD_{blank} is the standard deviation of the method blanks. Similarly, the limits of detection (LODs) were calculated with the formula:

$$LOD = mean_{control} + t_{0.01,\ n-1} * SD_{control}$$

where $mean_{control}$ is the mean of control tissue measures, $t_{0.01,\ n-1}$ is Student's t-value for n – 1 degrees of freedom at the 99% confidence level, and $SD_{control}$ is the standard deviation of the control tissue measures.

Table S1. The canonical SMILES structure of all 209 PCB congeners. The molecular structure and IUPAC names were also listed for comparison.

EPA Name	Canonical SMILES	Molecular Formula	IUPAC Name
PCB1	C1=CC=C(C=C1)C2=CC=CC=C2Cl	C12H9Cl	2-Chloro-1,1'-biphenyl
PCB2	C1=CC=C(C=C1)C2=CC(=CC=C2)Cl	C12H9Cl	3-Chloro-1,1'-biphenyl
PCB3	C1=CC=C(C=C1)C2=CC=C(C=C2)Cl	C12H9Cl	4-Chloro-1,1'-biphenyl
PCB4	C1=CC=C(C(=C1)C2=CC=CC=C2Cl)Cl	C12H8Cl2	2,2'-Dichloro-1,1'-biphenyl
PCB5	C1=CC=C(C=C1)C2=C(C(=CC=C2)Cl)Cl	C12H8Cl2	2,3-Dichloro-1,1'-biphenyl
PCB6	C1=CC=C(C(=C1)C2=CC(=CC=C2)Cl)Cl	C12H8Cl2	2,3'-Dichloro-1,1'-biphenyl
PCB7	C1=CC=C(C=C1)C2=C(C=C(C=C2)Cl)Cl	C12H8Cl2	2,4-Dichloro-1,1'-biphenyl
PCB8	C1=CC=C(C(=C1)C2=CC=C(C=C2)Cl)Cl	C12H8Cl2	2,4'-Dichloro-1,1'-biphenyl
PCB9	C1=CC=C(C=C1)C2=C(C=CC(=C2)Cl)Cl	C12H8Cl2	2,5-Dichloro-1,1'-biphenyl
PCB10	C1=CC=C(C=C1)C2=C(C=CC=C2Cl)Cl	C12H8Cl2	2,6-Dichloro-1,1'-biphenyl
PCB11	C1=CC(=CC(=C1)Cl)C2=CC(=CC=C2)Cl	C12H8Cl2	3,3'-Dichloro-1,1'-biphenyl
PCB12	C1=CC=C(C=C1)C2=CC(=C(C=C2)Cl)Cl	C12H8Cl2	3,4-Dichloro-1,1'-biphenyl
PCB13	C1=CC(=CC(=C1)Cl)C2=CC=C(C=C2)Cl	C12H8Cl2	3,4'-Dichloro-1,1'-biphenyl
PCB14	C1=CC=C(C=C1)C2=CC(=CC(=C2)Cl)Cl	C12H8Cl2	3,5-Dichloro-1,1'-biphenyl
PCB15	C1=CC(=CC=C1C2=CC=C(C=C2)Cl)Cl	C12H8Cl2	4,4'-Dichloro-1,1'-biphenyl
PCB16	C1=CC=C(C(=C1)C2=C(C(=CC=C2)Cl)Cl)Cl	C12H7Cl3	2,2',3-Trichloro-1,1'-biphenyl
PCB17	C1=CC=C(C(=C1)C2=C(C=C(C=C2)Cl)Cl)Cl	C12H7Cl3	2,2',4-Trichloro-1,1'-biphenyl
PCB18	C1=CC=C(C(=C1)C2=C(C=CC(=C2)Cl)Cl)Cl	C12H7Cl3	2,2',5-Trichloro-1,1'-biphenyl
PCB19	C1=CC=C(C(=C1)C2=C(C=CC=C2Cl)Cl)Cl	C12H7Cl3	2,2',6-Trichloro-1,1'-biphenyl
PCB20	C1=CC(=CC(=C1)Cl)C2=C(C(=CC=C2)Cl)Cl	C12H7Cl3	2,3,3'-Trichloro-1,1'-biphenyl
PCB21	C1=CC=C(C=C1)C2=C(C(=C(C=C2)Cl)Cl)Cl	C12H7Cl3	2,3,4-Trichloro-1,1'-biphenyl
PCB22	C1=CC(=C(C(=C1)Cl)Cl)C2=CC=C(C=C2)Cl	C12H7Cl3	2,3,4'-Trichloro-1,1'-biphenyl
PCB23	C1=CC=C(C=C1)C2=C(C(=CC(=C2)Cl)Cl)Cl	C12H7Cl3	2,3,5-Trichloro-1,1'-biphenyl
PCB24	C1=CC=C(C=C1)C2=C(C=CC(=C2Cl)Cl)Cl	C12H7Cl3	2,3,6-Trichloro-1,1'-biphenyl
PCB25	C1=CC(=CC(=C1)Cl)C2=C(C=C(C=C2)Cl)Cl	C12H7Cl3	2,3',4-Trichloro-1,1'-biphenyl
PCB26	C1=CC(=CC(=C1)Cl)C2=C(C=CC(=C2)Cl)Cl	C12H7Cl3	2,3',5-Trichloro-1,1'-biphenyl
PCB27	C1=CC(=CC(=C1)Cl)C2=C(C=CC=C2Cl)Cl	C12H7Cl3	2,3',6-Trichloro-1,1'-biphenyl
PCB28	C1=CC(=CC=C1C2=C(C=C(C=C2)Cl)Cl)Cl	C12H7Cl3	2,4,4'-Trichloro-1,1'-biphenyl
PCB29	C1=CC=C(C=C1)C2=CC(=C(C=C2Cl)Cl)Cl	C12H7Cl3	2,4,5-Trichloro-1,1'-biphenyl
PCB30	C1=CC=C(C=C1)C2=C(C=C(C=C2Cl)Cl)Cl	C12H7Cl3	2,4,6-Trichloro-1,1'-biphenyl

EPA Name	Canonical SMILES	Molecular Formula	IUPAC Name
PCB31	C1=CC(=CC=C1C2=C(C=CC(=C2)Cl)Cl)Cl	C12H7Cl3	2,4',5-Trichloro-1,1'-biphenyl
PCB32	C1=CC(=C(C(=C1)Cl)C2=CC=C(C=C2)Cl)Cl	C12H7Cl3	2,4',6-Trichloro-1,1'-biphenyl
PCB33	C1=CC=C(C(=C1)C2=CC(=C(C=C2)Cl)Cl)Cl	C12H7Cl3	2,3',4'-Trichloro-1,1'-biphenyl
PCB34	C1=CC=C(C(=C1)C2=CC(=CC(=C2)Cl)Cl)Cl	C12H7Cl3	2,3',5'-Trichloro-1,1'-biphenyl
PCB35	C1=CC(=CC(=C1)Cl)C2=CC(=C(C=C2)Cl)Cl	C12H7Cl3	3,3',4-Trichloro-1,1'-biphenyl
PCB36	C1=CC(=CC(=C1)Cl)C2=CC(=CC(=C2)Cl)Cl	C12H7Cl3	3,3',5-Trichloro-1,1'-biphenyl
PCB37	C1=CC(=CC=C1C2=CC(=C(C=C2)Cl)Cl)Cl	C12H7Cl3	3,4,4'-Trichloro-1,1'-biphenyl
PCB38	C1=CC=C(C=C1)C2=CC(=C(C(=C2)Cl)Cl)Cl	C12H7Cl3	3,4,5-Trichloro-1,1'-biphenyl
PCB39	C1=CC(=CC=C1C2=CC(=CC(=C2)Cl)Cl)Cl	C12H7Cl3	3,4',5-Trichloro-1,1'-biphenyl
PCB40	C1=CC(=C(C(=C1)Cl)Cl)C2=C(C(=CC=C2)Cl)Cl	C12H6Cl4	2,2',3,3'-Tetrachloro-1,1'-biphenyl
PCB41	C1=CC=C(C(=C1)C2=C(C(=C(C=C2)Cl)Cl)Cl)Cl	C12H6Cl4	2,2',3,4-Tetrachloro-1,1'-biphenyl
PCB42	C1=CC(=C(C(=C1)Cl)Cl)C2=C(C=C(C=C2)Cl)Cl	C12H6Cl4	2,2',3,4'-Tetrachloro-1,1'-biphenyl
PCB43	C1=CC=C(C(=C1)C2=C(C(=CC(=C2)Cl)Cl)Cl)Cl	C12H6Cl4	2,2',3,5-Tetrachloro-1,1'-biphenyl
PCB44	C1=CC(=C(C(=C1)Cl)Cl)C2=C(C=CC(=C2)Cl)Cl	C12H6Cl4	2,2',3,5'-Tetrachloro-1,1'-biphenyl
PCB45	C1=CC=C(C(=C1)C2=C(C=CC(=C2Cl)Cl)Cl)Cl	C12H6Cl4	2,2',3,6-Tetrachloro-1,1'-biphenyl
PCB46	C1=CC(=C(C(=C1)Cl)Cl)C2=C(C=CC=C2Cl)Cl	C12H6Cl4	2,2',3,6'-Tetrachloro-1,1'-biphenyl
PCB47	C1=CC(=C(C=C1Cl)Cl)C2=C(C=C(C=C2)Cl)Cl	C12H6Cl4	2,2',4,4'-Tetrachloro-1,1'-biphenyl
PCB48	C1=CC=C(C(=C1)C2=CC(=C(C=C2Cl)Cl)Cl)Cl	C12H6Cl4	2,2',4,5-Tetrachloro-1,1'-biphenyl
PCB49	C1=CC(=C(C=C1Cl)Cl)C2=C(C=CC(=C2)Cl)Cl	C12H6Cl4	2,2',4,5'-Tetrachloro-1,1'-biphenyl
PCB50	C1=CC=C(C(=C1)C2=C(C=C(C=C2Cl)Cl)Cl)Cl	C12H6Cl4	2,2',4,6-Tetrachloro-1,1'-biphenyl
PCB51	C1=CC(=C(C(=C1)Cl)C2=C(C=C(C=C2)Cl)Cl)Cl	C12H6Cl4	2,2',4,6'-Tetrachloro-1,1'-biphenyl
PCB52	C1=CC(=C(C=C1Cl)C2=C(C=CC(=C2)Cl)Cl)Cl	C12H6Cl4	2,2',5,5'-Tetrachloro-1,1'-biphenyl
PCB53	C1=CC(=C(C(=C1)Cl)C2=C(C=CC(=C2)Cl)Cl)Cl	C12H6Cl4	2,2',5,6'-Tetrachloro-1,1'-biphenyl
PCB54	C1=CC(=C(C(=C1)Cl)C2=C(C=CC=C2Cl)Cl)Cl	C12H6Cl4	2,2',6,6'-Tetrachloro-1,1'-biphenyl
PCB55	C1=CC(=CC(=C1)Cl)C2=C(C(=C(C=C2)Cl)Cl)Cl	C12H6Cl4	2,3,3',4-Tetrachloro-1,1'-biphenyl
PCB56	C1=CC(=C(C(=C1)Cl)Cl)C2=CC(=C(C=C2)Cl)Cl	C12H6Cl4	2,3,3',4'-Tetrachloro-1,1'-biphenyl
PCB57	C1=CC(=CC(=C1)Cl)C2=C(C(=CC(=C2)Cl)Cl)Cl	C12H6Cl4	2,3,3',5-Tetrachloro-1,1'-biphenyl
PCB58	C1=CC(=C(C(=C1)Cl)Cl)C2=CC(=CC(=C2)Cl)Cl	C12H6Cl4	2,3,3',5'-Tetrachloro-1,1'-biphenyl
PCB59	C1=CC(=CC(=C1)Cl)C2=C(C=CC(=C2Cl)Cl)Cl	C12H6Cl4	2,3,3',6-Tetrachloro-1,1'-biphenyl
PCB60	C1=CC(=CC=C1C2=C(C(=C(C=C2)Cl)Cl)Cl)Cl	C12H6Cl4	2,3,4,4'-Tetrachloro-1,1'-biphenyl
PCB61	C1=CC=C(C=C1)C2=CC(=C(C(=C2Cl)Cl)Cl)Cl	C12H6Cl4	2,3,4,5-Tetrachloro-1,1'-biphenyl
PCB62	C1=CC=C(C=C1)C2=C(C(=C(C=C2Cl)Cl)Cl)Cl	C12H6Cl4	2,3,4,6-Tetrachloro-1,1'-biphenyl
PCB63	C1=CC(=CC=C1C2=C(C(=CC(=C2)Cl)Cl)Cl)Cl	C12H6Cl4	2,3,4',5-Tetrachloro-1,1'-biphenyl

EPA Name	Canonical SMILES	Molecular Formula	IUPAC Name
PCB64	C1=CC(=CC=C1C2=C(C=CC(=C2Cl)Cl)Cl)Cl	C12H6Cl4	2,3,4',6-Tetrachloro-1,1'-biphenyl
PCB65	C1=CC=C(C=C1)C2=C(C(=CC(=C2Cl)Cl)Cl)Cl	C12H6Cl4	2,3,5,6-Tetrachloro-1,1'-biphenyl
PCB66	C1=CC(=C(C=C1C2=C(C=C(C=C2)Cl)Cl)Cl)Cl	C12H6Cl4	2,3',4,4'-Tetrachloro-1,1'-biphenyl
PCB67	C1=CC(=CC(=C1)Cl)C2=CC(=C(C=C2Cl)Cl)Cl	C12H6Cl4	2,3',4,5-Tetrachloro-1,1'-biphenyl
PCB68	C1=CC(=C(C=C1Cl)Cl)C2=CC(=CC(=C2)Cl)Cl	C12H6Cl4	2,3',4,5'-Tetrachloro-1,1'-biphenyl
PCB69	C1=CC(=CC(=C1)Cl)C2=C(C=C(C=C2Cl)Cl)Cl	C12H6Cl4	2,3',4,6-Tetrachloro-1,1'-biphenyl
PCB70	C1=CC(=C(C=C1C2=C(C=CC(=C2)Cl)Cl)Cl)Cl	C12H6Cl4	2,3',4',5-Tetrachloro-1,1'-biphenyl
PCB71	C1=CC(=C(C(=C1)Cl)C2=CC(=C(C=C2)Cl)Cl)Cl	C12H6Cl4	2,3',4',6-Tetrachloro-1,1'-biphenyl
PCB72	C1=CC(=C(C=C1Cl)C2=CC(=CC(=C2)Cl)Cl)Cl	C12H6Cl4	2,3',5,5'-Tetrachloro-1,1'-biphenyl
PCB73	C1=CC(=C(C(=C1)Cl)C2=CC(=CC(=C2)Cl)Cl)Cl	C12H6Cl4	2,3',5',6-Tetrachloro-1,1'-biphenyl
PCB74	C1=CC(=CC=C1C2=CC(=C(C=C2Cl)Cl)Cl)Cl	C12H6Cl4	2,4,4',5-Tetrachloro-1,1'-biphenyl
PCB75	C1=CC(=CC=C1C2=C(C=C(C=C2Cl)Cl)Cl)Cl	C12H6Cl4	2,4,4',6-Tetrachloro-1,1'-biphenyl
PCB76	C1=CC=C(C(=C1)C2=CC(=C(C(=C2)Cl)Cl)Cl)Cl	C12H6Cl4	2,3',4',5'-Tetrachloro-1,1'-biphenyl
PCB77	C1=CC(=C(C=C1C2=CC(=C(C=C2)Cl)Cl)Cl)Cl	C12H6Cl4	3,3',4,4'-Tetrachloro-1,1'-biphenyl
PCB78	C1=CC(=CC(=C1)Cl)C2=CC(=C(C(=C2)Cl)Cl)Cl	C12H6Cl4	3,3',4,5-Tetrachloro-1,1'-biphenyl
PCB79	C1=CC(=C(C=C1C2=CC(=CC(=C2)Cl)Cl)Cl)Cl	C12H6Cl4	3,3',4,5'-Tetrachloro-1,1'-biphenyl
PCB80	C1=C(C=C(C=C1Cl)Cl)C2=CC(=CC(=C2)Cl)Cl	C12H6Cl4	3,3',5,5'-Tetrachloro-1,1'-biphenyl
PCB81	C1=CC(=CC=C1C2=CC(=C(C(=C2)Cl)Cl)Cl)Cl	C12H6Cl4	3,4,4',5-Tetrachloro-1,1'-biphenyl
PCB82	C1=CC(=C(C(=C1)Cl)Cl)C2=C(C(=C(C=C2)Cl)Cl)Cl	C12H5Cl5	2,2',3,3',4-Pentachloro-1,1'-biphenyl
PCB83	C1=CC(=C(C(=C1)Cl)Cl)C2=C(C(=CC(=C2)Cl)Cl)Cl	C12H5Cl5	2,2',3,3',5-Pentachloro-1,1'-biphenyl
PCB84	C1=CC(=C(C(=C1)Cl)Cl)C2=C(C=CC(=C2Cl)Cl)Cl	C12H5Cl5	2,2',3,3',6-Pentachloro-1,1'-biphenyl
PCB85	C1=CC(=C(C=C1Cl)Cl)C2=C(C(=C(C=C2)Cl)Cl)Cl	C12H5Cl5	2,2',3,4,4'-Pentachloro-1,1'-biphenyl
PCB86	C1=CC=C(C(=C1)C2=CC(=C(C(=C2Cl)Cl)Cl)Cl)Cl	C12H5Cl5	2,2',3,4,5-Pentachloro-1,1'-biphenyl
PCB87	C1=CC(=C(C=C1Cl)C2=C(C(=C(C=C2)Cl)Cl)Cl)Cl	C12H5Cl5	2,2',3,4,5'-Pentachloro-1,1'-biphenyl
PCB88	C1=CC=C(C(=C1)C2=C(C(=C(C=C2Cl)Cl)Cl)Cl)Cl	C12H5Cl5	2,2',3,4,6-Pentachloro-1,1'-biphenyl
PCB89	C1=CC(=C(C(=C1)Cl)C2=C(C(=C(C=C2)Cl)Cl)Cl)Cl	C12H5Cl5	2,2',3,4,6'-Pentachloro-1,1'-biphenyl
PCB90	C1=CC(=C(C=C1Cl)Cl)C2=C(C(=CC(=C2)Cl)Cl)Cl	C12H5Cl5	2,2',3,4',5-Pentachloro-1,1'-biphenyl
PCB91	C1=CC(=C(C=C1Cl)Cl)C2=C(C=CC(=C2Cl)Cl)Cl	C12H5Cl5	2,2',3,4',6-Pentachloro-1,1'-biphenyl
PCB92	C1=CC(=C(C=C1Cl)C2=C(C(=CC(=C2)Cl)Cl)Cl)Cl	C12H5Cl5	2,2',3,5,5'-Pentachloro-1,1'-biphenyl
PCB93	C1=CC=C(C(=C1)C2=C(C(=CC(=C2Cl)Cl)Cl)Cl)Cl	C12H5Cl5	2,2',3,5,6-Pentachloro-1,1'-biphenyl
PCB94	C1=CC(=C(C(=C1)Cl)C2=C(C(=CC(=C2)Cl)Cl)Cl)Cl	C12H5Cl5	2,2',3,5,6'-Pentachloro-1,1'-biphenyl
PCB95	C1=CC(=C(C=C1Cl)C2=C(C=CC(=C2Cl)Cl)Cl)Cl	C12H5Cl5	2,2',3,5',6-Pentachloro-1,1'-biphenyl
PCB96	C1=CC(=C(C(=C1)Cl)C2=C(C=CC(=C2Cl)Cl)Cl)Cl	C12H5Cl5	2,2',3,6,6'-Pentachloro-1,1'-biphenyl

EPA Name	Canonical SMILES	Molecular Formula	IUPAC Name
PCB97	C1=CC(=C(C(=C1)Cl)Cl)C2=CC(=C(C=C2Cl)Cl)Cl	C12H5Cl5	2,2',3,4',5'-Pentachloro-1,1'-biphenyl
PCB98	C1=CC(=C(C(=C1)Cl)Cl)C2=C(C=C(C=C2Cl)Cl)Cl	C12H5Cl5	2,2',3,4',6'-Pentachloro-1,1'-biphenyl
PCB99	C1=CC(=C(C=C1Cl)Cl)C2=CC(=C(C=C2Cl)Cl)Cl	C12H5Cl5	2,2',4,4',5-Pentachloro-1,1'-biphenyl
PCB100	C1=CC(=C(C=C1Cl)Cl)C2=C(C=C(C=C2Cl)Cl)Cl	C12H5Cl5	2,2',4,4',6-Pentachloro-1,1'-biphenyl
PCB101	C1=CC(=C(C=C1Cl)C2=CC(=C(C=C2Cl)Cl)Cl)Cl	C12H5Cl5	2,2',4,5,5'-Pentachloro-1,1'-biphenyl
PCB102	C1=CC(=C(C(=C1)Cl)C2=CC(=C(C=C2Cl)Cl)Cl)Cl	C12H5Cl5	2,2',4,5,6'-Pentachloro-1,1'-biphenyl
PCB103	C1=CC(=C(C=C1Cl)C2=C(C=C(C=C2Cl)Cl)Cl)Cl	C12H5Cl5	2,2',4,5',6-Pentachloro-1,1'-biphenyl
PCB104	C1=CC(=C(C(=C1)Cl)C2=C(C=C(C=C2Cl)Cl)Cl)Cl	C12H5Cl5	2,2',4,6,6'-Pentachloro-1,1'-biphenyl
PCB105	C1=CC(=C(C=C1C2=C(C(=C(C=C2)Cl)Cl)Cl)Cl)Cl	C12H5Cl5	2,3,3',4,4'-Pentachloro-1,1'-biphenyl
PCB106	C1=CC(=CC(=C1)Cl)C2=CC(=C(C(=C2Cl)Cl)Cl)Cl	C12H5Cl5	2,3,3',4,5-Pentachloro-1,1'-biphenyl
PCB107	C1=CC(=C(C=C1C2=C(C(=CC(=C2)Cl)Cl)Cl)Cl)Cl	C12H5Cl5	2,3,3',4',5-Pentachloro-1,1'-biphenyl
PCB108	C1=CC(=C(C(=C1C2=CC(=CC(=C2)Cl)Cl)Cl)Cl)Cl	C12H5Cl5	2,3,3',4,5'-Pentachloro-1,1'-biphenyl
PCB109	C1=CC(=CC(=C1)Cl)C2=C(C(=C(C=C2Cl)Cl)Cl)Cl	C12H5Cl5	2,3,3',4,6-Pentachloro-1,1'-biphenyl
PCB110	C1=CC(=C(C=C1C2=C(C=CC(=C2Cl)Cl)Cl)Cl)Cl	C12H5Cl5	2,3,3',4',6-Pentachloro-1,1'-biphenyl
PCB111	C1=C(C=C(C=C1Cl)Cl)C2=C(C(=CC(=C2)Cl)Cl)Cl	C12H5Cl5	2,3,3',5,5'-Pentachloro-1,1'-biphenyl
PCB112	C1=CC(=CC(=C1)Cl)C2=C(C(=CC(=C2Cl)Cl)Cl)Cl	C12H5Cl5	2,3,3',5,6-Pentachloro-1,1'-biphenyl
PCB113	C1=CC(=C(C(=C1Cl)C2=CC(=CC(=C2)Cl)Cl)Cl)Cl	C12H5Cl5	2,3,3',5',6-Pentachloro-1,1'-biphenyl
PCB114	C1=CC(=CC=C1C2=CC(=C(C(=C2Cl)Cl)Cl)Cl)Cl	C12H5Cl5	2,3,4,4',5-Pentachloro-1,1'-biphenyl
PCB115	C1=CC(=CC=C1C2=C(C(=C(C=C2Cl)Cl)Cl)Cl)Cl	C12H5Cl5	2,3,4,4',6-Pentachloro-1,1'-biphenyl
PCB116	C1=CC=C(C=C1)C2=C(C(=C(C(=C2Cl)Cl)Cl)Cl)Cl	C12H5Cl5	2,3,4,5,6-Pentachloro-1,1'-biphenyl
PCB117	C1=CC(=CC=C1C2=C(C(=CC(=C2Cl)Cl)Cl)Cl)Cl	C12H5Cl5	2,3,4',5,6-Pentachloro-1,1'-biphenyl
PCB118	C1=CC(=C(C=C1C2=CC(=C(C=C2Cl)Cl)Cl)Cl)Cl	C12H5Cl5	2,3',4,4',5-Pentachloro-1,1'-biphenyl
PCB119	C1=CC(=C(C=C1C2=C(C=C(C=C2Cl)Cl)Cl)Cl)Cl	C12H5Cl5	2,3',4,4',6-Pentachloro-1,1'-biphenyl
PCB120	C1=C(C=C(C=C1Cl)Cl)C2=CC(=C(C=C2Cl)Cl)Cl	C12H5Cl5	2,3',4,5,5'-Pentachloro-1,1'-biphenyl
PCB121	C1=C(C=C(C=C1Cl)Cl)C2=C(C=C(C=C2Cl)Cl)Cl	C12H5Cl5	2,3',4,5',6-Pentachloro-1,1'-biphenyl
PCB122	C1=CC(=C(C(=C1)Cl)Cl)C2=CC(=C(C(=C2)Cl)Cl)Cl	C12H5Cl5	2,3,3',4',5'-Pentachloro-1,1'-biphenyl
PCB123	C1=CC(=C(C=C1Cl)Cl)C2=CC(=C(C(=C2)Cl)Cl)Cl	C12H5Cl5	2,3',4,4',5'-Pentachloro-1,1'-biphenyl
PCB124	C1=CC(=C(C=C1Cl)C2=CC(=C(C(=C2)Cl)Cl)Cl)Cl	C12H5Cl5	2,3',4',5,5'-Pentachloro-1,1'-biphenyl
PCB125	C1=CC(=C(C(=C1)Cl)C2=CC(=C(C(=C2)Cl)Cl)Cl)Cl	C12H5Cl5	2,3',4',5',6-Pentachloro-1,1'-biphenyl
PCB126	C1=CC(=C(C=C1C2=CC(=C(C(=C2)Cl)Cl)Cl)Cl)Cl	C12H5Cl5	3,3',4,4',5-Pentachloro-1,1'-biphenyl
PCB127	C1=C(C=C(C=C1Cl)Cl)C2=CC(=C(C(=C2)Cl)Cl)Cl	C12H5Cl5	3,3',4,5,5'-Pentachloro-1,1'-biphenyl
PCB128	C1=CC(=C(C(=C1C2=C(C(=C(C=C2)Cl)Cl)Cl)Cl)Cl)Cl	C12H4Cl6	2,2',3,3',4,4'-Hexachloro-1,1'-biphenyl
PCB129	C1=CC(=C(C(=C1)Cl)Cl)C2=CC(=C(C(=C2Cl)Cl)Cl)Cl	C12H4Cl6	2,2',3,3',4,5-Hexachloro-1,1'-biphenyl

EPA Name	Canonical SMILES	Molecular Formula	IUPAC Name
PCB130	C1=CC(=C(C(=C1C2=C(C(=CC(=C2)Cl)Cl)Cl)Cl)Cl)Cl	C12H4Cl6	2,2',3,3',4,5'-Hexachloro-1,1'-biphenyl
PCB131	C1=CC(=C(C(=C1)Cl)Cl)C2=C(C(=C(C=C2Cl)Cl)Cl)Cl	C12H4Cl6	2,2',3,3',4,6-Hexachloro-1,1'-biphenyl
PCB132	C1=CC(=C(C(=C1C2=C(C=CC(=C2Cl)Cl)Cl)Cl)Cl)Cl	C12H4Cl6	2,2',3,3',4,6'-Hexachloro-1,1'-biphenyl
PCB133	C1=C(C=C(C(=C1C2=C(C(=CC(=C2)Cl)Cl)Cl)Cl)Cl)Cl	C12H4Cl6	2,2',3,3',5,5'-Hexachloro-1,1'-biphenyl
PCB134	C1=CC(=C(C(=C1)Cl)Cl)C2=C(C(=CC(=C2Cl)Cl)Cl)Cl	C12H4Cl6	2,2',3,3',5,6-Hexachloro-1,1'-biphenyl
PCB135	C1=CC(=C(C(=C1Cl)C2=C(C(=CC(=C2)Cl)Cl)Cl)Cl)Cl	C12H4Cl6	2,2',3,3',5,6'-Hexachloro-1,1'-biphenyl
PCB136	C1=CC(=C(C(=C1Cl)C2=C(C=CC(=C2Cl)Cl)Cl)Cl)Cl	C12H4Cl6	2,2',3,3',6,6'-Hexachloro-1,1'-biphenyl
PCB137	C1=CC(=C(C=C1Cl)Cl)C2=CC(=C(C(=C2Cl)Cl)Cl)Cl	C12H4Cl6	2,2',3,4,4',5-Hexachloro-1,1'-biphenyl
PCB138	C1=CC(=C(C(=C1C2=CC(=C(C=C2Cl)Cl)Cl)Cl)Cl)Cl	C12H4Cl6	2,2',3,4,4',5'-Hexachloro-1,1'-biphenyl
PCB139	C1=CC(=C(C=C1Cl)Cl)C2=C(C(=C(C=C2Cl)Cl)Cl)Cl	C12H4Cl6	2,2',3,4,4',6-Hexachloro-1,1'-biphenyl
PCB140	C1=CC(=C(C(=C1C2=C(C=C(C=C2Cl)Cl)Cl)Cl)Cl)Cl	C12H4Cl6	2,2',3,4,4',6'-Hexachloro-1,1'-biphenyl
PCB141	C1=CC(=C(C=C1Cl)C2=CC(=C(C(=C2Cl)Cl)Cl)Cl)Cl	C12H4Cl6	2,2',3,4,5,5'-Hexachloro-1,1'-biphenyl
PCB142	C1=CC=C(C(=C1)C2=C(C(=C(C(=C2Cl)Cl)Cl)Cl)Cl)Cl	C12H4Cl6	2,2',3,4,5,6-Hexachloro-1,1'-biphenyl
PCB143	C1=CC(=C(C(=C1)Cl)C2=CC(=C(C(=C2Cl)Cl)Cl)Cl)Cl	C12H4Cl6	2,2',3,4,5,6'-Hexachloro-1,1'-biphenyl
PCB144	C1=CC(=C(C=C1Cl)C2=C(C(=C(C=C2Cl)Cl)Cl)Cl)Cl	C12H4Cl6	2,2',3,4,5',6-Hexachloro-1,1'-biphenyl
PCB145	C1=CC(=C(C(=C1)Cl)C2=C(C(=C(C=C2Cl)Cl)Cl)Cl)Cl	C12H4Cl6	2,2',3,4,6,6'-Hexachloro-1,1'-biphenyl
PCB146	C1=C(C=C(C(=C1C2=CC(=C(C=C2Cl)Cl)Cl)Cl)Cl)Cl	C12H4Cl6	2,2',3,4',5,5'-Hexachloro-1,1'-biphenyl
PCB147	C1=CC(=C(C=C1Cl)Cl)C2=C(C(=CC(=C2Cl)Cl)Cl)Cl	C12H4Cl6	2,2',3,4',5,6-Hexachloro-1,1'-biphenyl
PCB148	C1=C(C=C(C(=C1C2=C(C=C(C=C2Cl)Cl)Cl)Cl)Cl)Cl	C12H4Cl6	2,2',3,4',5,6'-Hexachloro-1,1'-biphenyl
PCB149	C1=CC(=C(C(=C1Cl)C2=CC(=C(C=C2Cl)Cl)Cl)Cl)Cl	C12H4Cl6	2,2',3,4',5',6-Hexachloro-1,1'-biphenyl
PCB150	C1=CC(=C(C(=C1Cl)C2=C(C=C(C=C2Cl)Cl)Cl)Cl)Cl	C12H4Cl6	2,2',3,4',6,6'-Hexachloro-1,1'-biphenyl
PCB151	C1=CC(=C(C=C1Cl)C2=C(C(=CC(=C2Cl)Cl)Cl)Cl)Cl	C12H4Cl6	2,2',3,5,5',6-Hexachloro-1,1'-biphenyl
PCB152	C1=CC(=C(C(=C1)Cl)C2=C(C(=CC(=C2Cl)Cl)Cl)Cl)Cl	C12H4Cl6	2,2',3,5,6,6'-Hexachloro-1,1'-biphenyl
PCB153	C1=C(C(=CC(=C1Cl)Cl)Cl)C2=CC(=C(C=C2Cl)Cl)Cl	C12H4Cl6	2,2',4,4',5,5'-Hexachloro-1,1'-biphenyl
PCB154	C1=C(C=C(C(=C1Cl)C2=CC(=C(C=C2Cl)Cl)Cl)Cl)Cl	C12H4Cl6	2,2',4,4',5,6'-Hexachloro-1,1'-biphenyl
PCB155	C1=C(C=C(C(=C1Cl)C2=C(C=C(C=C2Cl)Cl)Cl)Cl)Cl	C12H4Cl6	2,2',4,4',6,6'-Hexachloro-1,1'-biphenyl
PCB156	C1=CC(=C(C=C1C2=CC(=C(C(=C2Cl)Cl)Cl)Cl)Cl)Cl	C12H4Cl6	2,3,3',4,4',5-Hexachloro-1,1'-biphenyl
PCB157	C1=CC(=C(C(=C1C2=CC(=C(C(=C2)Cl)Cl)Cl)Cl)Cl)Cl	C12H4Cl6	2,3,3',4,4',5'-Hexachloro-1,1'-biphenyl
PCB158	C1=CC(=C(C=C1C2=C(C(=C(C=C2Cl)Cl)Cl)Cl)Cl)Cl	C12H4Cl6	2,3,3',4,4',6-Hexachloro-1,1'-biphenyl
PCB159	C1=C(C=C(C=C1Cl)Cl)C2=CC(=C(C(=C2Cl)Cl)Cl)Cl	C12H4Cl6	2,3,3',4,5,5'-Hexachloro-1,1'-biphenyl
PCB160	C1=CC(=CC(=C1)Cl)C2=C(C(=C(C(=C2Cl)Cl)Cl)Cl)Cl	C12H4Cl6	2,3,3',4,5,6-Hexachloro-1,1'-biphenyl
PCB161	C1=C(C=C(C=C1Cl)Cl)C2=C(C(=C(C=C2Cl)Cl)Cl)Cl	C12H4Cl6	2,3,3',4,5',6-Hexachloro-1,1'-biphenyl
PCB162	C1=C(C=C(C(=C1Cl)Cl)Cl)C2=C(C(=CC(=C2)Cl)Cl)Cl	C12H4Cl6	2,3,3',4',5,5'-Hexachloro-1,1'-biphenyl

EPA Name	Canonical SMILES	Molecular Formula	IUPAC Name
PCB163	C1=CC(=C(C=C1C2=C(C(=CC(=C2Cl)Cl)Cl)Cl)Cl)Cl	C12H4Cl6	2,3,3',4',5,6-Hexachloro-1,1'-biphenyl
PCB164	C1=CC(=C(C(=C1Cl)C2=CC(=C(C(=C2)Cl)Cl)Cl)Cl)Cl	C12H4Cl6	2,3,3',4',5',6-Hexachloro-1,1'-biphenyl
PCB165	C1=C(C=C(C=C1Cl)Cl)C2=C(C(=CC(=C2Cl)Cl)Cl)Cl	C12H4Cl6	2,3,3',5,5',6-Hexachloro-1,1'-biphenyl
PCB166	C1=CC(=CC=C1C2=C(C(=C(C(=C2Cl)Cl)Cl)Cl)Cl)Cl	C12H4Cl6	2,3,4,4',5,6-Hexachloro-1,1'-biphenyl
PCB167	C1=C(C=C(C(=C1Cl)Cl)Cl)C2=CC(=C(C=C2Cl)Cl)Cl	C12H4Cl6	2,3',4,4',5,5'-Hexachloro-1,1'-biphenyl
PCB168	C1=C(C=C(C(=C1Cl)Cl)Cl)C2=C(C=C(C=C2Cl)Cl)Cl	C12H4Cl6	2,3',4,4',5',6-Hexachloro-1,1'-biphenyl
PCB169	C1=C(C=C(C(=C1Cl)Cl)Cl)C2=CC(=C(C(=C2)Cl)Cl)Cl	C12H4Cl6	3,3',4,4',5,5'-Hexachloro-1,1'-biphenyl
PCB170	C1=CC(=C(C(=C1C2=CC(=C(C(=C2Cl)Cl)Cl)Cl)Cl)Cl)Cl	C12H3Cl7	2,2',3,3',4,4',5-Heptachloro-1,1'-biphenyl
PCB171	C1=CC(=C(C(=C1C2=C(C(=C(C=C2Cl)Cl)Cl)Cl)Cl)Cl)Cl	C12H3Cl7	2,2',3,3',4,4',6-Heptachloro-1,1'-biphenyl
PCB172	C1=C(C=C(C(=C1C2=CC(=C(C(=C2Cl)Cl)Cl)Cl)Cl)Cl)Cl	C12H3Cl7	2,2',3,3',4,5,5'-Heptachloro-1,1'-biphenyl
PCB173	C1=CC(=C(C(=C1)Cl)Cl)C2=C(C(=C(C(=C2Cl)Cl)Cl)Cl)Cl	C12H3Cl7	2,2',3,3',4,5,6-Heptachloro-1,1'-biphenyl
PCB174	C1=CC(=C(C(=C1Cl)C2=CC(=C(C(=C2Cl)Cl)Cl)Cl)Cl)Cl	C12H3Cl7	2,2',3,3',4,5,6'-Heptachloro-1,1'-biphenyl
PCB175	C1=C(C=C(C(=C1C2=C(C(=C(C=C2Cl)Cl)Cl)Cl)Cl)Cl)Cl	C12H3Cl7	2,2',3,3',4,5',6-Heptachloro-1,1'-biphenyl
PCB176	C1=CC(=C(C(=C1Cl)C2=C(C(=C(C=C2Cl)Cl)Cl)Cl)Cl)Cl	C12H3Cl7	2,2',3,3',4,6,6'-Heptachloro-1,1'-biphenyl
PCB177	C1=CC(=C(C(=C1C2=C(C(=CC(=C2Cl)Cl)Cl)Cl)Cl)Cl)Cl	C12H3Cl7	2,2',3,3',4,5',6'-Heptachloro-1,1'-biphenyl
PCB178	C1=C(C=C(C(=C1C2=C(C(=CC(=C2Cl)Cl)Cl)Cl)Cl)Cl)Cl	C12H3Cl7	2,2',3,3',5,5',6-Heptachloro-1,1'-biphenyl
PCB179	C1=CC(=C(C(=C1Cl)C2=C(C(=CC(=C2Cl)Cl)Cl)Cl)Cl)Cl	C12H3Cl7	2,2',3,3',5,6,6'-Heptachloro-1,1'-biphenyl
PCB180	C1=C(C(=CC(=C1Cl)Cl)Cl)C2=CC(=C(C(=C2Cl)Cl)Cl)Cl	C12H3Cl7	2,2',3,4,4',5,5'-Heptachloro-1,1'-biphenyl
PCB181	C1=CC(=C(C=C1Cl)Cl)C2=C(C(=C(C(=C2Cl)Cl)Cl)Cl)Cl	C12H3Cl7	2,2',3,4,4',5,6-Heptachloro-1,1'-biphenyl
PCB182	C1=C(C=C(C(=C1Cl)C2=CC(=C(C(=C2Cl)Cl)Cl)Cl)Cl)Cl	C12H3Cl7	2,2',3,4,4',5,6'-Heptachloro-1,1'-biphenyl
PCB183	C1=C(C(=CC(=C1Cl)Cl)Cl)C2=C(C(=C(C=C2Cl)Cl)Cl)Cl	C12H3Cl7	2,2',3,4,4',5',6-Heptachloro-1,1'-biphenyl
PCB184	C1=C(C=C(C(=C1Cl)C2=C(C(=C(C=C2Cl)Cl)Cl)Cl)Cl)Cl	C12H3Cl7	2,2',3,4,4',6,6'-Heptachloro-1,1'-biphenyl
PCB185	C1=CC(=C(C=C1Cl)C2=C(C(=C(C(=C2Cl)Cl)Cl)Cl)Cl)Cl	C12H3Cl7	2,2',3,4,5,5',6-Heptachloro-1,1'-biphenyl
PCB186	C1=CC(=C(C(=C1)Cl)C2=C(C(=C(C(=C2Cl)Cl)Cl)Cl)Cl)Cl	C12H3Cl7	2,2',3,4,5,6,6'-Heptachloro-1,1'-biphenyl
PCB187	C1=C(C(=CC(=C1Cl)Cl)Cl)C2=C(C(=CC(=C2Cl)Cl)Cl)Cl	C12H3Cl7	2,2',3,4',5,5',6-Heptachloro-1,1'-biphenyl
PCB188	C1=C(C=C(C(=C1Cl)C2=C(C(=CC(=C2Cl)Cl)Cl)Cl)Cl)Cl	C12H3Cl7	2,2',3,4',5,6,6'-Heptachloro-1,1'-biphenyl
PCB189	C1=C(C=C(C(=C1Cl)Cl)Cl)C2=CC(=C(C(=C2Cl)Cl)Cl)Cl	C12H3Cl7	2,3,3',4,4',5,5'-Heptachloro-1,1'-biphenyl
PCB190	C1=CC(=C(C=C1C2=C(C(=C(C(=C2Cl)Cl)Cl)Cl)Cl)Cl)Cl	C12H3Cl7	2,3,3',4,4',5,6-Heptachloro-1,1'-biphenyl
PCB191	C1=C(C=C(C(=C1Cl)Cl)Cl)C2=C(C(=C(C=C2Cl)Cl)Cl)Cl	C12H3Cl7	2,3,3',4,4',5',6-Heptachloro-1,1'-biphenyl
PCB192	C1=C(C=C(C=C1Cl)Cl)C2=C(C(=C(C(=C2Cl)Cl)Cl)Cl)Cl	C12H3Cl7	2,3,3',4,5,5',6-Heptachloro-1,1'-biphenyl
PCB193	C1=C(C=C(C(=C1Cl)Cl)Cl)C2=C(C(=CC(=C2Cl)Cl)Cl)Cl	C12H3Cl7	2,3,3',4',5,5',6-Heptachloro-1,1'-biphenyl
PCB194	C1=C(C(=C(C(=C1Cl)Cl)Cl)Cl)C2=CC(=C(C(=C2Cl)Cl)Cl)Cl	C12H2Cl8	2,2',3,3',4,4',5,5'-Octachloro-1,1'-biphenyl
PCB195	C1=CC(=C(C(=C1C2=C(C(=C(C(=C2Cl)Cl)Cl)Cl)Cl)Cl)Cl)Cl	C12H2Cl8	2,2',3,3',4,4',5,6-Octachloro-1,1'-biphenyl

EPA Name	Canonical SMILES	Molecular Formula	IUPAC Name
PCB196	C1=C(C(=C(C(=C1Cl)Cl)Cl)Cl)C2=C(C(=C(C=C2Cl)Cl)Cl)Cl	C12H2Cl8	2,2',3,3',4,4',5,6'-Octachloro-1,1'-biphenyl
PCB197	C1=C(C(=C(C(=C1Cl)Cl)Cl)Cl)C2=C(C(=C(C=C2Cl)Cl)Cl)Cl)Cl	C12H2Cl8	2,2',3,3',4,4',6,6'-Octachloro-1,1'-biphenyl
PCB198	C1=C(C=C(C(=C1C2=C(C(=C(C(=C2Cl)Cl)Cl)Cl)Cl)Cl)Cl)Cl	C12H2Cl8	2,2',3,3',4,5,5',6-Octachloro-1,1'-biphenyl
PCB199	C1=C(C(=C(C(=C1Cl)Cl)Cl)Cl)C2=C(C(=CC(=C2Cl)Cl)Cl)Cl	C12H2Cl8	2,2',3,3',4,5,5',6'-Octachloro-1,1'-biphenyl
PCB200	C1=CC(=C(C(=C1Cl)C2=C(C(=C(C(=C2Cl)Cl)Cl)Cl)Cl)Cl)Cl	C12H2Cl8	2,2',3,3',4,5,6,6'-Octachloro-1,1'-biphenyl
PCB201	C1=C(C(=C(C(=C1Cl)Cl)Cl)C2=C(C(=CC(=C2Cl)Cl)Cl)Cl)Cl	C12H2Cl8	2,2',3,3',4,5',6,6'-Octachloro-1,1'-biphenyl
PCB202	C1=C(C(=C(C(=C1Cl)Cl)C2=C(C(=CC(=C2Cl)Cl)Cl)Cl)Cl)Cl	C12H2Cl8	2,2',3,3',5,5',6,6'-Octachloro-1,1'-biphenyl
PCB203	C1=C(C(=CC(=C1Cl)Cl)Cl)C2=C(C(=C(C(=C2Cl)Cl)Cl)Cl)Cl	C12H2Cl8	2,2',3,4,4',5,5',6-Octachloro-1,1'-biphenyl
PCB204	C1=C(C=C(C(=C1Cl)C2=C(C(=C(C(=C2Cl)Cl)Cl)Cl)Cl)Cl)Cl	C12H2Cl8	2,2',3,4,4',5,6,6'-Octachloro-1,1'-biphenyl
PCB205	C1=C(C=C(C(=C1Cl)Cl)Cl)C2=C(C(=C(C(=C2Cl)Cl)Cl)Cl)Cl	C12H2Cl8	2,3,3',4,4',5,5',6-Octachloro-1,1'-biphenyl
PCB206	C1=C(C(=C(C(=C1Cl)Cl)Cl)Cl)C2=C(C(=C(C(=C2Cl)Cl)Cl)Cl)Cl	C12HCl9	2,2',3,3',4,4',5,5',6-Nonachloro-1,1'-biphenyl
PCB207	C1=C(C(=C(C(=C1Cl)Cl)Cl)C2=C(C(=C(C(=C2Cl)Cl)Cl)Cl)Cl)Cl	C12HCl9	2,2',3,3',4,4',5,6,6'-Nonachloro-1,1'-biphenyl
PCB208	C1=C(C(=C(C(=C1Cl)Cl)C2=C(C(=C(C(=C2Cl)Cl)Cl)Cl)Cl)Cl)Cl	C12HCl9	2,2',3,3',4,5,5',6,6'-Nonachloro-1,1'-biphenyl
PCB209	C1(=C(C(=C(C(=C1Cl)Cl)Cl)Cl)Cl)C2=C(C(=C(C(=C2Cl)Cl)Cl) Cl)Cl	C12Cl10	2,2',3,3',4,4',5,5',6,6'-Decachloro-1,1'-biphenyl

The table was generated from a link created by the Iowa Superfund Research Program (ISRP) and was accessible from https://apps.iowasuperfund.uiowa.edu/pcb-ids/

Table S2. The Canonical SMILES structure of 124 available methoxyl-PCBs used in calibration standards as derivative forms for corresponding OH-PCBs.

Compound ID	Compound name	Chlorine pattern	MeO pattern	Canonical SMILES
meopcb001	2-MeO-PCB3	4	2	ClC(C=C1OC)=CC=C1C2=CC=CC=C2
meopcb002	2'-MeO-PCB3	4	2'	ClC(C=C1)=CC=C1C2=C(OC)C=CC=C2
meopcb003	3-MeO-PCB3	4	3	ClC(C(OC)=C1)=CC=C1C2=CC=CC=C2
meopcb004	2-MeO-PCB11	3,3'	2	ClC1=C(OC)C(C2=CC(Cl)=CC=C2)=CC=C1
meopcb005	4,5-diMeO-PCB1	2	4,5	ClC1=CC(OC)=C(OC)C=C1C2=CC=CC=C2
meopcb006	2',3'-diMeO-PCB3	4	2',3'	ClC(C=C1)=CC=C1C2=C(OC)C(OC)=CC=C2
meopcb007	6-MeO-PCB11	3,3'	6	ClC1=CC=C(OC)C(C2=CC=CC(Cl)=C2)=C1
meopcb008	2',3'-diMeO-PCB5	2,3	2',3'	ClC1=C(Cl)C(C2=C(OC)C(OC)=CC=C2)=CC=C1
meopcb009	4'-MeO-PCB5	2,3	4'	ClC1=C(Cl)C(C2=CC=C(OC)C=C2)=CC=C1
meopcb010	2'-MeO-PCB28	2,4,4'	2'	ClC1=CC(Cl)=CC=C1C2=C(OC)C=C(Cl)C=C2
meopcb011	2',5'-diMeO-PCB8	2,4'	2'5'	ClC1=CC=CC=C1C2=C(OC)C=C(Cl)C(OC)=C2
meopcb012	4,5-diMeO-PCB4	2,2'	4,5	ClC1=CC(OC)=C(OC)C=C1C2=C(Cl)C=CC=C2
meopcb013	5-MeO-PCB11	3,3'	5	ClC1=CC(OC)=CC(C2=CC=CC(Cl)=C2)=C1
meopcb014	3-MeO-PCB50	2,2',4,6	3	ClC(C(OC)=C1Cl)=CC(Cl)=C1C2=C(Cl)C=CC=C2
meopcb015	2',5'-diMeO-PCB5	2,3	2',5'	ClC1=C(Cl)C=CC=C1C2=C(OC)C=CC(OC)=C2
meopcb016	3',4'-diMeO-PCB3	4	3',4'	ClC(C=C1)=CC=C1C2=CC=C(OC)C(OC)=C2
meopcb017	5,6-diMeO-PCB11	3,3'	5,6	ClC1=CC(OC)=C(OC)C(C2=CC=CC(Cl)=C2)=C1
meopcb018	2,5-diMeO-PCB11	3,3'	2,5	ClC1=CC(OC)=CC(C2=CC=CC(Cl)=C2)=C1OC
meopcb019	4-MeO-PCB11	3,3'	4	ClC1=C(OC)C=CC(C2=CC=CC(Cl)=C2)=C1
meopcb020	3-MeO-PCB28	2,4,4'	3	ClC1=C(OC)C(Cl)=C(C2=CC=C(Cl)C=C2)C=C1
meopcb021	2',3'-diMeO-PCB12	3,4	2',3'	ClC(C(Cl)=C1)=CC=C1C2=C(OC)C(OC)=CC=C2
meopcb022	2',3'-diMeO-PCB9	2,5	2',3'	ClC1=CC(C2=C(OC)C(OC)=CC=C2)=C(Cl)C=C1
meopcb023	4'-MeO-PCB12	3,4	4'	ClC1=CC(C2=CC=C(OC)C=C2)=CC=C1Cl
meopcb024	4'-MeO-PCB35	3,3',4	4'	ClC1=CC(C2=CC(Cl)=C(Cl)C=C2)=CC=C1OC
meopcb025	2',5'-MeO-PCB14	3,5	2,5'	ClC1=CC(C2=CC(OC)=CC=C2OC)=CC(Cl)=C1
meopcb026	3'-MeO-PCB28	2,4,4'	3'	ClC1=CC(Cl)=C(C2=CC=C(Cl)C(OC)=C2)C=C1
meopcb027	5-MeO-PCB28	2,4,4'	5	ClC1=CC(Cl)=C(C2=CC=C(Cl)C=C2)C=C1OC
meopcb028	3',4'-diMeO-PCB5	2,3	3',4'	ClC1=C(Cl)C=CC=C1C2=CC=C(OC)C(OC)=C2
meopcb029	4,5-diMeO-PCB8	2,4'	4,5	ClC1=CC(OC)=C(OC)C=C1C2=CC=C(Cl)C=C2
meopcb030	2,5-diMeO-PCB15	4,4'	2,5	ClC(C=C1)=CC=C1C2=CC(OC)=C(Cl)C=C2OC

Compound ID	Compound name	Chlorine pattern	MeO pattern	Canonical SMILES
meopcb031	4'-MeO-PCB29	2,4,5	4'	ClC1=C(Cl)C=C(C2=CC=C(OC)C=C2)C(Cl)=C1
meopcb032	4-MeO-PCB36	3,3',5	4	ClC1=C(OC)C(Cl)=CC(C2=CC=CC(Cl)=C2)=C1
meopcb033	4'-MeO-PCB25	2,3'4	4'	ClC1=CC(Cl)=C(C2=CC=C(OC)C(Cl)=C2)C=C1
meopcb034	4,5-diMeO-PCB11	2,4'	4,5	ClC1=C(OC)C(OC)=CC(C2=CC=CC(Cl)=C2)=C1
meopcb035	2',5'-diMeO-PCB31	2,4',5	2',5'	ClC(C(OC)=C1)=CC(OC)=C1C2=C(Cl)C=CC(Cl)=C2
meopcb036	3-MeO-PCB103	2,2',4,5',6	3	ClC1=CC(C2=C(Cl)C(OC)=C(Cl)C=C2Cl)=C(Cl)C=C1
meopcb037	3',4'-diMeO-PCB14	3,5	3',4'	ClC1=CC(C2=CC(OC)=C(OC)C=C2)=CC(Cl)=C1
meopcb038	3-MeO-PCB100	2,2',4,4',6	3	ClC1=C(C2=C(Cl)C=C(Cl)C=C2)C(Cl)=CC(Cl)=C1OC
meopcb039	4'-MeO-PCB68	2,3',4,5'	4'	ClC1=C(C2=CC(Cl)=C(OC)C(Cl)=C2)C=CC(Cl)=C1
meopcb040	3'-MeO-PCB98	2,2',3,4',6'	3'	ClC1=C(C2=C(Cl)C=C(Cl)C(OC)=C2Cl)C=CC=C1Cl
meopcb041	3',4'-diMeO-PCB12	3,4	3',4'	ClC1=CC=C(C2=CC(OC)=C(OC)C=C2)C=C1Cl
meopcb042	3'-MeO-PCB150	2,2',3,4',6,6'	3'	ClC1=C(C2=C(Cl)C=C(Cl)C(OC)=C2Cl)C(Cl)=CC=C1Cl
meopcb043	4'-MeO-PCB95	2,2',3,5',6	4'	ClC1=C(C2=CC(Cl)=C(OC)C=C2Cl)C(Cl)=CC=C1Cl
meopcb044	5-MeO-PCB91	2,2',3,4',6	5	ClC1=C(C2=CC=C(Cl)C=C2Cl)C(Cl)=C(OC)C=C1Cl
meopcb045	4,5-diMeO-PCB95	2,2',3,5',6	4,5	ClC1=C(C2=CC(Cl)=CC=C2Cl)C(Cl)=C(OC)C(OC)=C1Cl
meopcb046	4-MeO-PCB91	2,2',3,4',6	4	ClC1=C(C2=CC=C(Cl)C=C2Cl)C(Cl)=CC(OC)=C1Cl
meopcb047	4,5-diMeO-PCB91	2,2',3,4',6	4,5	ClC1=C(C2=CC=C(Cl)C=C2Cl)C(Cl)=C(OC)C(OC)=C1Cl
meopcb048	4,5-diMeO-PCB136	2,2',3,3',6,6'	4,5	ClC1=C(C2=C(Cl)C=CC(Cl)=C2Cl)C(Cl)=C(OC)C(OC)=C1Cl
meopcb049	4-MeO-PCB136	2,2',3,3',6,6'	4	ClC1=C(C2=C(Cl)C=CC(Cl)=C2Cl)C(Cl)=CC(OC)=C1Cl
meopcb050	3'-MeO-PCB140	2,2',3,4,4',6'	3'	ClC1=C(C2=C(Cl)C=C(Cl)C(OC)=C2Cl)C=CC(Cl)=C1Cl
meopcb051	4,4'-diMeO-PCB52	2,2',5,5'	4,4'	ClC1=C(C2=CC(Cl)=C(OC)C=C2Cl)C=C(Cl)C(OC)=C1
meopcb052	4,5-diMeO-PCB132	2,2',3,3',4,6'	4,5	ClC1=C(C2=C(Cl)C=C(Cl)C(Cl)=C2Cl)C=C(OC)C(OC)=C1Cl
meopcb053	2-MeO-PCB2	3	2	ClC1=CC=CC(C2=CC=CC=C2)=C1OC
meopcb054	2'-MeO-PCB2	3	2'	ClC1=CC=CC(C2=CC=CC=C2OC)=C1
meopcb055	6-MeO-PCB2	3	6	ClC1=CC=C(OC)C(C2=CC=CC=C2)=C1
meopcb056	4-MeO-PCB1	2	4	ClC1=CC(OC)=CC=C1C2=CC=CC=C2
meopcb057	5-MeO-PCB2	3	5	ClC1=CC(OC)=CC(C2=CC=CC=C2)=C1
meopcb058	3'-MeO-PCB2	3	3'	ClC1=CC=CC(C2=CC=CC(OC)=C2)=C1
meopcb059	2'-MeO-PCB5	2,3	2'	ClC1=C(C=CC=C1C2=C(C=CC=C2)OC)Cl
meopcb060	4-MeO-PCB2	3	4	ClC1=C(OC)C=CC(C2=CC=CC=C2)=C1
meopcb061	4'-MeO-PCB2	3	4'	ClC1=CC=CC(C2=CC=C(OC)C=C2)=C1
meopcb062	4'-MeO-PCB3	4	4'	ClC1=CC=C(C2=CC=C(C=C2)OC)C=C1
meopcb063	2'-MeO-PCB30	2,4,6	2'	ClC1=CC(Cl)=CC(Cl)=C1C2=CC=CC=C2OC

Compound ID	Compound name	Chlorine pattern	MeO pattern	Canonical SMILES
meopcb064	3'-MeO-PCB9	2,5	3'	ClC1=CC(C2=CC(OC)=CC=C2)=C(Cl)C=C1
meopcb065	2'-MeO-PCB12	3,4	2'	ClC(C(Cl)=C1)=CC=C1C2=C(OC)C=CC=C2
meopcb066	4-MeO-PCB14	3,5	4	ClC1=CC(C2=CC=CC=C2)=CC(Cl)=C1OC
meopcb067	4'-MeO-PCB9	2,5	4'	ClC1=CC(C2=CC=C(OC)C=C2)=C(Cl)C=C1
meopcb068	3'-MeO-PCB30	2,4,6	3'	ClC1=CC(Cl)=CC(Cl)=C1C2=CC=CC(OC)=C2
meopcb069	6'-MeO-PCB26	2,3',5	6'	ClC1=C(C2=C(OC)C=CC(Cl)=C2)C=C(Cl)C=C1
meopcb070	4'-MeO-PCB18	2,2',5	4'	ClC1=C(C2=CC=C(OC)C=C2Cl)C=C(Cl)C=C1
meopcb071	4'-MeO-PCB30	2,4,6	4'	ClC1=CC(Cl)=CC(Cl)=C1C2=CC=C(OC)C=C2
meopcb072	3-MeO-PCB54	2,2',6,6'	3	ClC1=C(C2=C(Cl)C=CC=C2Cl)C(Cl)=CC=C1OC
meopcb073	6'-MeO-PCB69	2,3',4,6	6'	ClC1=C(C2=C(OC)C=CC(Cl)=C2)C(Cl)=CC(Cl)=C1
meopcb074	2'-MeO-PCB65	2,3,5,6	2'	ClC1=C(C2=CC=CC=C2OC)C(Cl)=C(Cl)C=C1Cl
meopcb075	4'-MeO-PCB26	2,3',5	4'	ClC1=C(C=C(C=C1)Cl)C2=CC=C(OC)C(Cl)=C2
meopcb076	4-MeO-PCB65	2,3,5,6	4	ClC1=C(C2=CC=CC=C2)C(Cl)=C(Cl)C(OC)=C1Cl
meopcb077	3'-MeO-PCB65	2,3,5,6	3'	ClC1=C(C2=CC=CC(OC)=C2)C(Cl)=C(Cl)C=C1Cl
meopcb078	2'-MeO-PCB61	2,3,4,5	2'	ClC1=C(C2=CC=CC=C2OC)C=C(Cl)C(Cl)=C1Cl
meopcb079	6'-MeO-PCB101	2,2',4,5,5'	6'	ClC1=C(C2=C(OC)C(Cl)=CC=C2Cl)C=C(Cl)C(Cl)=C1
meopcb080	4'-MeO-PCB72	2,3',5,5'	4'	ClC1=C(C2=CC(Cl)=C(OC)C(Cl)=C2)C=C(Cl)C=C1
meopcb081	4'-MeO-PCB69	2,3',4,6	4'	ClC1=C(C2=CC=C(OC)C(Cl)=C2)C(Cl)=CC(Cl)=C1
meopcb082	4'-MeO-PCB65	2,3,5,6	4'	ClC1=C(C2=CC=C(OC)C=C2)C(Cl)=C(Cl)C=C1Cl
meopcb083	6'-MeO-PCB83	2,2',3,3',5	6'	ClC1=C(C2=C(OC)C=CC(Cl)=C2Cl)C=C(Cl)C=C1Cl
meopcb084	3'-MeO-PCB61	2,3,4,5	3'	ClC1=C(C2=CC=CC(OC)=C2)C=C(Cl)C(Cl)=C1Cl
meopcb085	4'-MeO-PCB93	2,2',3,5,6	4'	ClC1=C(C2=CC=C(OC)C=C2Cl)C(Cl)=C(Cl)C=C1Cl
meopcb086	4'-MeO-PCB61	2,3,4,5	4'	ClC1=C(C2=CC=C(OC)C=C2)C=C(Cl)C(Cl)=C1Cl
meopcb087	4'-MeO-PCB79	3,3',4,5'	4'	ClC1=C(Cl)C=CC(C2=CC(Cl)=C(OC)C(Cl)=C2)=C1
meopcb088	4'-MeO-PCB101	2,2',4,5,5'	4'	ClC1=C(C2=CC(Cl)=C(OC)C=C2Cl)C=C(Cl)C(Cl)=C1
meopcb089	2'-MeO-PCB106	2,3,3',4,5	2'	ClC1=C(Cl)C(Cl)=CC(C2=CC=CC(Cl)=C2OC)=C1Cl
meopcb090	2'-MeO-PCB114	2,3,4,4',5	2'	ClC1=C(Cl)C(Cl)=CC(C2=CC=C(Cl)C=C2OC)=C1Cl
meopcb091	3,3'di-MeO-PCB155	2,2',4,4',6,6'	3,3'	ClC1=C(OC)C(Cl)=C(C2=C(Cl)C=C(Cl)C(OC)=C2Cl)C(Cl)=C1
meopcb092	4'-MeO-PCB120	2,3',4,5,5'	4'	ClC1=CC(Cl)=C(C2=CC(Cl)=C(OC)C(Cl)=C2)C=C1Cl
meopcb093	4-MeO-PCB134	2,2',3,3',5,6	4	ClC1=C(C2=CC=CC(Cl)=C2Cl)C(Cl)=C(Cl)C(OC)=C1Cl
meopcb094	4'-MeO-PCB86	2,2',3,4,5	4'	ClC1=C(Cl)C(Cl)=C(C2=CC=C(OC)C=C2Cl)C=C1Cl
meopcb095	4-MeO-PCB97	2,2',3,4',5'	4	ClC1=C(C2=CC(Cl)=C(Cl)C=C2Cl)C=CC(OC)=C1Cl
meopcb096	4'-MeO-PCB108	2,3,3',4,5'	4'	ClC1=C(Cl)C(Cl)=C(C2=CC(Cl)=C(OC)C(Cl)=C2)C=C1

Compound ID	Compound name	Chlorine pattern	MeO pattern	Canonical SMILES
meopcb097	3-MeO-PCB118	2,3',4,4',5	3	ClC1=C(OC)C(Cl)=C(C2=CC=C(Cl)C(Cl)=C2)C=C1Cl
meopcb098	4-MeO-PCB107	2,3,3',4',5	4	ClC1=C(C2=CC=C(Cl)C(Cl)=C2)C=C(Cl)C(OC)=C1Cl
meopcb099	3'-MeO-PCB184	2,2',3,4,4',6,6'	3'	ClC1=C(Cl)C(Cl)=C(C2=C(Cl)C=C(Cl)C(OC)=C2Cl)C(Cl)=C1
meopcb100	4-MeO-PCB146	2,2',3,4',5,5'	4	ClC1=C(C2=CC(Cl)=C(Cl)C=C2Cl)C=C(Cl)C(OC)=C1Cl
meopcb101	3'-MeO-PCB138	2,2',3,4,4',5'	3'	ClC1=C(Cl)C(Cl)=C(C2=CC(Cl)=C(Cl)C(OC)=C2Cl)C=C1
meopcb102	4'-MeO-PCB130	2,2',3,3',4,5'	4'	ClC1=C(Cl)C(Cl)=C(C2=CC(Cl)=C(OC)C(Cl)=C2Cl)C=C1
meopcb103	4'-MeO-PCB127	3,3',4,5,5'	4'	ClC1=C(Cl)C=C(C2=CC(Cl)=C(OC)C(Cl)=C2)C=C1Cl
meopcb104	4-MeO-PCB178	2,2',3,3',5,5',6	4	ClC1=C(OC)C(Cl)=C(Cl)C(C2=CC(Cl)=CC(Cl)=C2Cl)=C1Cl
meopcb105	4-MeO-PCB163	2,3,3',4',5,6	4	ClC1=C(OC)C(Cl)=C(Cl)C(C2=CC=C(Cl)C(Cl)=C2)=C1Cl
meopcb106	3'-MeO-PCB182	2,2',3,4,4',5,6'	3'	ClC1=C(Cl)C(Cl)=C(C2=C(Cl)C=C(Cl)C(OC)=C2Cl)C=C1Cl
meopcb107	3'-MeO-PCB183	2,2',3,4,4',5',6	3'	ClC1=C(Cl)C(Cl)=C(C2=CC(Cl)=C(Cl)C(OC)=C2Cl)C(Cl)=C1
meopcb108	5-MeO-PCB183	2,2',3,4,4',5',6	5	ClC1=C(Cl)C(Cl)=C(C2=CC(Cl)=C(Cl)C=C2Cl)C(Cl)=C1OC
meopcb109	4-MeO-PCB187	2,2',3,4',5,5',6	4	ClC1=C(OC)C(Cl)=C(Cl)C(C2=CC(Cl)=C(Cl)C=C2Cl)=C1Cl
meopcb110	5-MeO-PCB138	2,2',3,4,4',5'	5	ClC1=C(Cl)C(Cl)=C(C2=CC(Cl)=C(Cl)C=C2Cl)C=C1OC
meopcb111	4-MeO-PCB202	2,2',3,3',5,5',6,6'	4	ClC1=C(OC)C(Cl)=C(Cl)C(C2=C(Cl)C(Cl)=CC(Cl)=C2Cl)=C1Cl
meopcb112	4'-MeO-PCB177	2,2',3,3',4,5',6'	4'	ClC1=C(Cl)C(Cl)=C(C2=C(Cl)C(Cl)=C(OC)C(Cl)=C2Cl)C=C1
meopcb113	4'-MeO-PCB159	2,3',3',4,5,5'	4'	ClC1=C(Cl)C(Cl)=C(C2=CC(Cl)=C(OC)C(Cl)=C2)C=C1Cl
meopcb114	4-MeO-PCB162	2,3,3',4',5,5'	4	ClC1=C(OC)C(Cl)=CC(C2=CC(Cl)=C(Cl)C(Cl)=C2)=C1Cl
meopcb115	4'-MeO-PCB201	2,2',3,3',4,5',6,6'	4'	ClC1=C(Cl)C(Cl)=C(C2=C(Cl)C(Cl)=C(OC)C(Cl)=C2Cl)C(Cl)=C1
meopcb116	4-MeO-PCB193	2,3,3',4',5,5',6	4	ClC1=C(OC)C(Cl)=C(Cl)C(C2=CC(Cl)=C(Cl)C(Cl)=C2)=C1Cl
meopcb117	3'-MeO-PCB180	2,2'3,4,4',5,5'	3'	ClC1=C(Cl)C(Cl)=C(C2=CC(Cl)=C(Cl)C(OC)=C2Cl)C=C1Cl
meopcb118	4'-MeO-PCB172	2,2',3,3',4,5,5'	4'	ClC1=C(Cl)C(Cl)=C(C2=CC(Cl)=C(OC)C(Cl)=C2Cl)C=C1Cl
meopcb119	4,4'di-MeO-PCB202	2,2',3,3',5,5',6,6'	4,4'	ClC1=C(OC)C(Cl)=C(Cl)C(C2=C(Cl)C(Cl)=C(OC)C(Cl)=C2Cl)=C1Cl
meopcb120	4'-MeO-PCB198	2,2',3,3',4,5,5',6	4'	ClC1=C(Cl)C(Cl)=C(C2=CC(Cl)=C(OC)C(Cl)=C2Cl)C(Cl)=C1Cl
meopcb121	4'-MeO-PCB200	2,2',3,3',4,5,6,6'	4'	ClC1=C(Cl)C(Cl)=C(C2=C(Cl)C=C(OC)C(Cl)=C2Cl)C(Cl)=C1Cl
meopcb122	3'-MeO-PCB203	2,2',3,4,4',5,5',6	3'	ClC1=C(Cl)C(Cl)=C(C2=CC(Cl)=C(Cl)C(OC)=C2Cl)C(Cl)=C1Cl
meopcb123	4'-MeO-PCB199	2,2',3,3',4,5,5',6'	4'	ClC1=C(Cl)C(Cl)=C(C2=C(Cl)C(Cl)=C(OC)C(Cl)=C2Cl)C=C1Cl
meopcb124	4'-MeO-PCB208	2,2',3,3',4,5,5',6,6'	4'	ClC1=C(Cl)C(Cl)=C(C2=C(Cl)C(Cl)=C(OC)C(Cl)=C2Cl)C(Cl)=C1Cl

Table S3. The body mass of animals used in this study.

	**Male**				**Female**			
	**0 mg/kg**	**0.1 mg/kg**	**1 mg/kg**	**6 mg/kg**	**0 mg/kg**	**0.1 mg/kg**	**1 mg/kg**	**6 mg/kg**
Body Mass (g)	20.7 ± 1.4	21.4 ± 1.6	21.2 ± 1.7	21.6 ± 2.4	16.9 ± 1.3	17.5 ± 1.4	17.4 ± 1.5	16.7 ± 1.0

Mean ± standard deviation. Not significant as assessed by Kruskal-Wallis test, male p=0.6, female p=0.3

Table S4. Precursor ions, product ions, and collision energies for each analyte in the GC-MS/MS analysis.

Analyte	Precursor Ion (*m/z*)	Product Ion (*m/z*)	Collision Energy (eV)
4'-9	252	209	20
PCB11	222	152	25
2-11	252	202	25
4-11	252	209	25
5-11	252	222	20
6-11	252	202	25
5,6-11	282	232	25
2,5-11	282	232	25
4,5-11	282	204	25
PCB15	222	152	25
PCB28	256	186	25
d-PCB30	261	191	30
2'-28	286	236	25
3-28	286	243	25
3'-28	286	243	25
5-28	286	243	25
4'-25	286	243	25
PCB52	292	222	25
4-52	322	279	20
4,4'-52	352	337	20
PCB84	326	256	25
4-91	356	313	25
PCB95	326	256	25
4-95	356	313	25
4'-95	356	313	25
5-95	356	313	25
4,5-95	386	343	25
3-103	356	306	25
PCB101	326	256	25
4'-101	356	313	25
6'-101	356	306	25
PCB117	326	256	25
PCB118	326	256	20
3-118	356	313	25
PCB135	360	290	25
PCB138	360	290	25
3'-138	390	347	25
5-138	390	347	25
PCB149	360	290	25
PCB153	360	290	25

**Analyte**	**Precursor Ion (*m/z*)**	**Product Ion (*m/z*)**	**Collision Energy (eV)**
3-153	390	347	25
4-146	390	347	25
4'-159	390	375	15
PCB180	394	323	25
3'-180	424	381	25
PCB204	430	358	25

Table S5. Recoveries (%) of surrogate standards for PCB and OH-PCB in different biological matrices. Values are mean ± standard deviation.

Surrogate Standards	Bladder (n=28)	Blood (n=28)	Urine (n=28)	Liver (n=20)
PCB15	93 ± 9	85 ± 12	75 ± 16	72 ± 4
4'-OH-PCB9	59 ± 12	61 ± 10	72 ± 9	74 ± 7
PCB117	99 ± 5	108 ± 13	86 ± 15	83 ± 7
4-OH-PCB91	97 ± 9	106 ± 15	89 ± 15	84 ± 7
4'-OH-PCB159	81 ±5	107 ± 14	101 ± 16	91 ± 8

Table S6. Ongoing Precision Recovery (OPR) for selected PCBs and OH-PCBs in method blanks and tissue matrices. Values are mean ± standard deviation (n = 7).

PCB/OH-PCB	Recovery in method blank (%)	Recovery in tissue matrices (%)
PCB11	71 ± 8	90 ± 7
PCB15	71 ± 5	90 ± 11
PCB28	81 ± 3	110 ± 16
4'-OH-PCB9	77 ± 18	72 ± 11
PCB52	85 ± 5	100 ± 7
4-OH-PCB11	84 ± 30	71 ± 27
PCB95	88 ± 7	99 ± 7
PCB84	90 ± 9	101 ± 6
4'-OH-PCB25	92 ± 34	79 ± 31
PCB101	91 ± 9	103 ± 7
4-OH-PCB52	89 ± 33	80 ± 30
PCB117	94 ± 12	107 ± 7
PCB135	90 ± 6	96 ± 9
PCB149	91 ± 7	96 ± 9
5-OH-PCB95	94 ± 38	85 ± 33
PCB118	100 ± 15	117 ± 11
4-OH-PCB95	93 ± 36	88 ± 33
4-OH-PCB91	106 ± 18	105 ± 11
PCB153	92 ± 8	102 ± 11
PCB138	95 ± 8	100 ± 9
3-OH-PCB153	100 ± 14	99 ± 9
4-OH-PCB146	98 ± 11	97 ± 9
PCB180	104 ± 10	109 ± 14
4'-OH-PCB159	94 ± 12	93 ± 8

Table S7. Method Detection Limits (MDLs) and Limits of Detection (LODs) for PCBs from MARBLES mixture and their possible OH-PCB metabolites.

PCB/OH-PCB	MDL[a] (ng) (n=14)	LOD[b] (ng/g)			
		Urine (n=6)	Blood (n=6)	Bladder (n=8)	Liver (n=8)
PCB 11	0.080	0.150	0.070	6.400	<0.001
2-OH-PCB 11	<0.001	<0.001	<0.001	<0.001	0.060
4-OH-PCB 11	<0.001	<0.001	<0.001	<0.001	<0.001
5-OH-PCB 11	0.080	<0.001	0.300	<0.001	<0.001
6-OH-PCB 11	<0.001	<0.001	<0.001	<0.001	<0.001
5,6-OH-PCB 11	0.001	0.003	0.003	<0.001	<0.001
2,5-OH-PCB 11	0.001	0.003	0.005	<0.001	<0.001
4,5-OH-PCB 11	<0.001	<0.001	<0.001	<0.001	<0.001
PCB 28	0.100	0.510	<0.001	6.340	1.250
2'-OH-PCB 28	0.004	0.020	0.006	0.150	0.020
3-OH-PCB 28	<0.001	0.020	<0.001	<0.001	<0.001
3'-OH-PCB 28	<0.001	0.130	<0.001	<0.001	0.110
5-OH-PCB 28	<0.001	0.100	<0.001	<0.001	<0.001
4'-OH-PCB 25[c]	<0.001	<0.001	<0.001	<0.001	0.070
PCB 52	0.110	0.210	0.200	2.140	9.150
4-OH-PCB 52	0.090	0.250	0.050	1.490	<0.001
4,4'-OH-PCB 52	<0.001	<0.001	<0.001	<0.001	<0.001
PCB 84	0.060	0.050	0.030	0.900	1.970
PCB 95	0.110	0.110	0.210	2.970	12.230
4-OH-PCB 95	0.070	<0.001	0.170	<0.001	<0.001
4'-OH-PCB 95	<0.001	<0.001	<0.001	<0.001	<0.001
5-OH-PCB 95	0.020	<0.001	0.020	<0.001	<0.001
4,5-OH-PCB 95	<0.001	<0.001	<0.001	<0.001	<0.001
3-OH-PCB 103[c]	0.001	<0.001	0.004	0.280	<0.001
PCB 101	0.150	0.260	0.250	3.030	13.070
4'-OH-PCB 101	<0.001	<0.001	<0.001	<0.001	<0.001
6'-OH-PCB 101	0.007	<0.001	0.010	<0.001	<0.001
PCB 118	0.110	0.570	0.100	6.740	5.680
3-OH-PCB 118	<0.001	<0.001	<0.001	<0.001	<0.001
PCB 135	0.080	0.030	0.050	1.060	2.360
PCB 138	0.120	0.700	0.070	3.140	6.260
3'-OH-PCB 138	<0.001	<0.001	<0.001	<0.001	<0.001
5-OH-PCB 138	<0.001	<0.001	<0.001	<0.001	0.006

**PCB/OH-PCB**	**MDL[a] (ng) (n=14)**	**LOD[b] (ng/g)**			
		**Urine (n=6)**	**Blood (n=6)**	**Bladder (n=8)**	**Liver (n=8)**
PCB 149	0.090	0.190	0.070	1.310	8.340
PCB 153	0.100	0.570	0.060	8.220	5.810
3-OH-PCB 153	0.040	0.030	0.050	0.440	<0.001
4-OH-PCB 146[c]	0.040	0.040	0.030	0.910	0.230
PCB 180	0.060	0.420	0.050	<0.001	0.560
3'-OH-PCB 180	<0.001	<0.001	<0.001	<0.001	0.030

[a] MDL, Method Detection Limits (ng) were calculated using the formula: MDL= $mean_{blank} + t_{0.01, n-1} * SD_{blank}$, where $mean_{blank}$ is the mean of method blanks, $t_{0.01, n-1}$ is Student's t-value for n – 1 degrees of freedom at the 99% confidence level, and SD_{blank} is the standard deviation of the method blanks. N = 14

[b] LOD, Limit of Detection (ng/g tissue weight) were adjusted by tissue mass and were calculated from formula: LOD= $mean_{control} + t_{0.01, n-1} * SD_{control}$, where $mean_{control}$ is the mean of control tissue measures, $t_{0.01, n-1}$ is Student's t-value for n – 1 degrees of freedom at the 99% confidence level, and $SD_{control}$ is the standard deviation of the control tissue measures.

[c] Possible metabolites due to NIH shift.

Table S8. RT-qPCR Primer information.

Gene	Tissue	Annealing Temperature (°C)/Notes on Primer concentration	Forward	Reverse	Potential additional products
Ppia	Bladder	60	CATACAGGTCCTGGCATCTTG	TCATGCCTTCTTTCACCTTCC	
Cyp4x1	Bladder	58	GAGCAACTCTGGCATTGGTTC	CTTGGCTGAGTTTCTCTCTACGG	
Cyp4x1	Liver	64			
Cyp2s1	Bladder	66	GGCCACCTCTAATGTCGTCTG	CCATGGAGAGCTGATCCCTAAC	
Cyp2s1	Liver	64			
Ugt2b1	Bladder	61	CCATGGCATTCCTATTGTTGG	TTCAAGGCAGTGAGAAGGTCTG	
Ugt2b1	Liver	60			
Cyp1a1	Bladder	62; 1 ul primer	CAGCCTTCCCAAATGGTTTATG	ATGGACATGCAAGGACAGGAG	Transcript variant 1 and 2
Cyp1a1	Liver	60; 1ul primer			
Cyp1a2	Bladder	62; 1ul primer	CCAGCCCCTGCCCTTCAGTGGTA	TGGGAACCTGGGTCCTTGAGGC	
Cyp1a2	Liver	68			
Cyp2b10	Bladder	62	CCAAATCTCCAGGGCTCCAAGGC	TGCGGACTTGGGCTATTGGGAGG	
Cyp2b10	Liver	68			
Cyp2b9	Bladder	60; 1ul primer	CACCAGGACCCCATCCTCTA	TTTCTTGAAGCTGAATGAAACACT	*Cyp2b13* (1 base mismatch in each primer)
Cyp2b9	Liver	63	CTGAGACCACAAGCGCCAC	CTTGACCATGAGCAGGACTCC	
Cyp3a11	Bladder	64	ACAAGCAGGGATGGACCTGGTT	CCCATATCGGTAGAGGAGCACCA	
Cyp3a11	Liver	60; 1ul primer			
Cyp3a41	Bladder	60	GGCAGAAAGGTAGCCCTACAG	TGCCAGGAGGACCCATGTAT	*Cyp3a41a*, *Cyp3a41b*; *Cyp3a16* (1 base mismatch in each primer)
Cyp3a41	Liver	60			
Cyp3a13	Bladder	62	GTCATGGGTGAGTGGTTGCT	TCCCAGCTCCCAGGTTACAT	
Cyp3a16	Bladder	62	TGCAGTCATAACTGGAGCGT	ACCAGGCATCAAAATCAATCAGT	
Cyp3a16	Liver	60			
Pgk1	Liver	60	TGGTGAAAGCCACTTCTAGGG	CATGGCTGACTTTATCCTCTGTG	

Primer sequence and notes for each gene. Primers were diluted from a 100 μM stock to a 10 μM stock containing Forward and Reverse. Notes of 1 μl primer denote that 1 μL of this 10 μM forward and reverse stock was used in PCR reactions instead of 0.5 μL.

Table S9. PCB and OH-PCB levels in bladder from postnatal day P 47 ± 4 days pups exposed via the maternal diet to 0.1, 1, or 6 mg/kg body weight of the MARBLES PCB mixture (ng/g tissue, n=3 pools).

PCB/OH-PCB names	Male			Female		
	0.1 mg/kg	1 mg/kg	6 mg/kg	0.1 mg/kg	1 mg/kg	6 mg/kg
PCB11	--	--	--	--	--	--
2-OH-PCB11	--	--	--	--	--	--
PCB28	--	7±0	13±2	8±0	8±0	7±1
6-OH-PCB11	--	--	--	--	--	--
PCB52	--	--	--	--	--	--
2'-OH-PCB28	--	--	--	--	--	--
5-OH-PCB11	--	--	--	--	--	--
5,6-OH-PCB11	--	--	0.6±0.7	0.2±0.1	--	0.2±0.2
2,5-OH-PCB11	--	--	--	--	--	--
4-OH-PCB11	--	--	--	--	--	--
3-OH-PCB28	--	--	--	--	--	--
PCB95	--	--	--	--	--	--
PCB84	--	--	--	--	--	--
3'-OH-PCB28	--	--	--	--	--	--
5-OH-PCB28	--	--	--	--	--	--
4'-OH-PCB25	--	--	--	--	--	--
PCB101	--	--	--	--	--	--
4,5-OH-PCB11	--	--	--	--	--	--
3-OH-PCB103	--	--	--	--	--	--
4-OH-PCB52	--	--	--	--	--	--
6'-OH-PCB101	--	--	--	--	--	--
PCB135	--	--	--	--	--	--
PCB149	--	--	--	--	--	--
5-OH-PCB95	--	--	--	--	--	--
PCB118	35±23	120±40	570±450	25±17	180±70	490±290
4'-OH-PCB95	--	--	--	--	--	--
4-OH-PCB95	--	--	--	--	--	--
4,5-OH-PCB95	--	--	--	--	--	--
PCB153	42±33	160±30	700±500	26±20	220±60	520±260
PCB138	18±14	63±14	281±201	11±9	87±31	210±110
4'-OH-PCB101	--	--	--	--	--	--
4,4'-OH-PCB52	--	--	--	--	--	--
3-OH-PCB118	--	--	1.2±0.4	--	0.6±0.3	1.0±0.3
3-OH-PCB153	--	--	0.9±0.3	--	0.5±0	0.8±0.2
4-OH-PCB146	--	--	--	--	--	--
3'-OH-PCB138	--	--	0.1±0	--	--	0.08±0.05
PCB180	99±71	300±60	680±320	220±10	500±110	550±180
5-OH-PCB138	--	--	--	--	--	--
3'-OH-PCB180	--	--	--	--	--	--
**Total**	190	640	2200	290	1000	1800

Values are mean ± standard deviation. --: below LOD (limit of detection).

Table S10. PCB and OH-PCB levels in the urine from postnatal day P 47 ± 4 days pups exposed via the maternal diet to 0.1, 1, or 6 mg/kg body weight of the MARBLES PCB mixture (ng/g urine, n=3 pools).

**PCB/OH-PCB names**	**Male**			**Female**		
	**0.1 mg/kg**	**1 mg/kg**	**6 mg/kg**	**0.1 mg/kg**	**1 mg/kg**	**6 mg/kg**
PCB11	--	--	--	--	--	--
2-OH-PCB11	--	--	--	--	--	--
PCB28	2.1±1.5	12.2±9.8	14.5±4.0	0.8±0.3	3.9±3.3	3.7±2.5
6-OH-PCB11	--	--	--	--	--	--
PCB52	--	--	--	--	--	--
2'-OH-PCB28	--	--	--	--	0.02±0	0.02±0
5-OH-PCB11	--	--	--	--	--	--
5,6-OH-PCB11	--	--	--	--	--	--
2,5-OH-PCB11	--	--	--	--	--	--
4-OH-PCB11	--	--	--	--	--	--
3-OH-PCB28	0.02±0	0.18±0.03	0.06±0.02	0.03±0	0.09±0.01	0.13±0.08
PCB95	--	--	--	--	0.12±0.01	--
PCB84	--	--	--	--	--	--
3'-OH-PCB28	0.30±0.03	2.5±0.7	0.89±0.47	0.27±0.11	1.6±0.3	1.8±1.4
5-OH-PCB28	0.14±0.01	1.0±0.2	0.33±0.14	0.23±0.10	1.3±0.4	1.3±0.9
4'-OH-PCB25	--	--	--	--	--	--
PCB101	--	--	0.30±0.03	--	--	--
4,5-OH-PCB11	--	--	--	--	--	--
3-OH-PCB103	--	--	--	--	--	--
4-OH-PCB52	--	--	--	--	--	--
6'-OH-PCB101	--	--	--	--	--	--
PCB135	--	--	--	--	0.04±0.01	--
PCB149	--	--	--	--	--	--
5-OH-PCB95	--	--	--	--	--	--
PCB118	1.1±0.5	4.8±2.2	25.7±5.5	--	3.6±3.2	6.7±3.4
4'-OH-PCB95	--	--	--	--	--	--
4-OH-PCB95	--	--	--	--	--	--
4,5-OH-PCB95	--	--	--	--	--	--
PCB153	0.74±0	3.0±0.5	17.1±2.5	--	1.6±1.1	1.7±0.9
PCB138	--	3.0±0.8	15.4±3.6	--	2.0±1.5	2.3±1.1
4'-OH-PCB101	--	--	--	--	--	--
4,4'-OH-PCB52	--	--	--	--	--	--
3-OH-PCB118	--	0.04±0.02	0.10±0.05	--	0.03±0.01	0.11±0.12
3-OH-PCB153	0.04±0	--	0.03±0	--	0.03±0	0.04±0.01
4-OH-PCB146	--	--	--	--	--	--
3'-OH-PCB138	0.01±0	0.003±0.002	0.008±0.001	--	--	--
PCB180	--	1.3±0.3	7.4±1.1	--	1.1±0.8	1.2±0.6
5-OH-PCB138	--	--	--	--	--	--
3'-OH-PCB180	--	--	--	--	--	--
**Total**	4.4	28	81	1.4	15	19

Values are mean ± standard deviation. --: below LOD (limit of detection).

Table S11. PCB and OH-PCB levels in the blood from postnatal day P 47 ± 4 days pups exposed via the maternal diet to 0.1, 1, or 6 mg/kg body weight of the MARBLES PCB mixture (ng/g blood, n=3 pools)

**PCB/OH-PCB names**	**Male**			**Female**		
	**0.1 mg/kg**	**1 mg/kg**	**6 mg/kg**	**0.1 mg/kg**	**1 mg/kg**	**6 mg/kg**
PCB11	--	--	--	--	--	--
2-OH-PCB11	--	--	--	--	--	--
PCB28	0.11±0.06	0.15±0.08	0.11±0.04	0.05±0.01	0.10±0.06	0.09±0.03
6-OH-PCB11	--	--	--	--	--	--
PCB52	--	--	--	--	--	--
2'-OH-PCB28	--	--	--	--	--	--
5-OH-PCB11	--	--	--	--	--	--
5,6-OH-PCB11	--	--	--	--	--	--
2,5-OH-PCB11	--	--	--	--	--	--
4-OH-PCB11	--	--	--	--	--	0.05±0.01
3-OH-PCB28	--	--	--	--	--	--
PCB95	--	--	--	--	--	--
PCB84	--	--	--	--	--	--
3'-OH-PCB28	--	--	--	--	0.03±0.03	--
5-OH-PCB28	--	--	--	--	--	--
4'-OH-PCB25	--	--	--	--	--	--
PCB101	--	--	--	--	--	0.34±0
4,5-OH-PCB11	--	--	--	--	--	--
3-OH-PCB103	--	--	--	--	--	--
4-OH-PCB52	--	--	--	--	--	--
6'-OH-PCB101	--	--	--	--	--	--
PCB135	--	--	--	--	--	--
PCB149	0.12±0.01	0.14±0.08	--	--	--	0.08±0
5-OH-PCB95	--	--	--	--	--	--
PCB118	0.25±0.08	2.5±0.5	5.6±2.2	0.35±0.14	2.5±0.5	8.6±2.7
4'-OH-PCB95	--	--	--	--	--	--
4-OH-PCB95	--	--	--	--	--	--
4,5-OH-PCB95	--	--	--	--	--	--
PCB153	0.52±0.20	5.1±1.7	7.7±2.9	0.34±0.15	2.7±0.2	10.2±4.5
PCB138	0.30±0.10	2.8±1.1	4.4±1.6	0.25±0.06	2.4±0.3	6.9±2.5
4'-OH-PCB101	--	--	--	--	--	--
4,4'-OH-PCB52	--	--	--	--	--	--
3-OH-PCB118	0.08±0.04	0.38±0.07	0.97±0.42	0.04±0.01	0.43±0.12	1.5±0.6
3-OH-PCB153	0.08±0.03	0.40±0.05	0.86±0.32	0.06±0.03	0.50±0.03	1.3±0.4
4-OH-PCB146	0.04±0	0.13±0.03	0.19±0.07	--	0.17±0.03	0.28±0.09
3'-OH-PCB138	0.02±0.01	0.07±0.01	0.13±0.05	0.007±0.004	0.07±0.01	0.18±0.07
PCB180	0.30±0.10	3.1±0.9	5.9±2.3	0.30±0.10	3.0±0.5	8.8±2.6
5-OH-PCB138	--	--	0.007±0.002	--	--	0.008±0.004
3'-OH-PCB180	--	0.04±0.01	0.08±0.03	0.005±0.002	0.06±0.01	0.15±0.04
**Total**	1.7	15	26	1.4	12	38

Values are mean ± standard deviation. --: below LOD (limit of detection).

Table S12. PCB and OH-PCB levels in the liver from postnatal day P 47 ± 4 days pups exposed via the maternal diet to 0.1, 1, or 6 mg/kg body weight of the MARBLES PCB mixture (ng/g wet tissue, n=4).

**PCB/OH-PCB names**	**Male**			**Female**		
	**0.1 mg/kg**	**1 mg/kg**	**6 mg/kg**	**0.1 mg/kg**	**1 mg/kg**	**6 mg/kg**
PCB11	--	--	--	--	--	--
2-OH-PCB11	--	--	--	--	--	--
PCB28	--	3.1±0.9	2.2±0.7	1.7±0.4	3.2±2.4	5.4±3.8
6-OH-PCB11	--	--	--	--	--	--
PCB52	--	--	--	--	--	--
2'-OH-PCB28	--	--	--	--	--	--
5-OH-PCB11	--	--	--	--	--	--
5,6-OH-PCB11	--	--	--	--	--	--
2,5-OH-PCB11	--	--	--	--	--	--
4-OH-PCB11	--	--	--	--	--	--
3-OH-PCB28	--	--	--	--	--	--
PCB95	--	--	--	--	--	--
PCB84	--	--	--	--	--	--
3'-OH-PCB28	--	--	--	--	--	--
5-OH-PCB28	--	--	--	--	--	--
4'-OH-PCB25	--	--	--	--	--	--
PCB101	--	--	--	--	--	--
4,5-OH-PCB11	--	--	--	--	--	--
3-OH-PCB103	--	--	--	--	--	--
4-OH-PCB52	--	--	--	--	--	--
6'-OH-PCB101	--	--	--	--	--	--
PCB135	--	--	--	--	--	--
PCB149	--	--	--	--	--	--
5-OH-PCB95	--	--	--	--	--	--
PCB118	6.6±0.9	65±24	170±80	11±3	95±62	320±10
4'-OH-PCB95	--	--	--	--	--	--
4-OH-PCB95	--	--	--	--	--	--
4,5-OH-PCB95	--	--	--	--	--	--
PCB153	7.4±1.2	78±29	260±130	12±3	120±88	380±60
PCB138	--	69±22	170±100	24±10	230±150	610±130
4'-OH-PCB101	--	--	--	--	--	--
4,4'-OH-PCB52	--	--	--	--	--	--
3-OH-PCB118	0.11±0.03	1.7±0.8	3.7±1.0	0.19±0.03	2.0±0.6	5.6±0.8
3-OH-PCB153	0.04±0.01	0.58±0.45	0.88±0.22	0.05±0.02	0.71±0.31	1.4±0.3
4-OH-PCB146	--	--	--	--	--	--
3'-OH-PCB138	0.01±0.00	0.18±0.08	0.34±0.10	0.01±0	0.18±0.02	0.40±0.09
PCB180	5.4±1.1	63±25	210±100	27±11	270±200	740±110
5-OH-PCB138	--	--	--	--	--	--
3'-OH-PCB180	--	--	0.08±0.05	--	0.07±0.03	0.13±0.06
**Total**	19	280	820	76	710	2100

Values are mean ± standard deviation. --: below LOD (limit of detection).

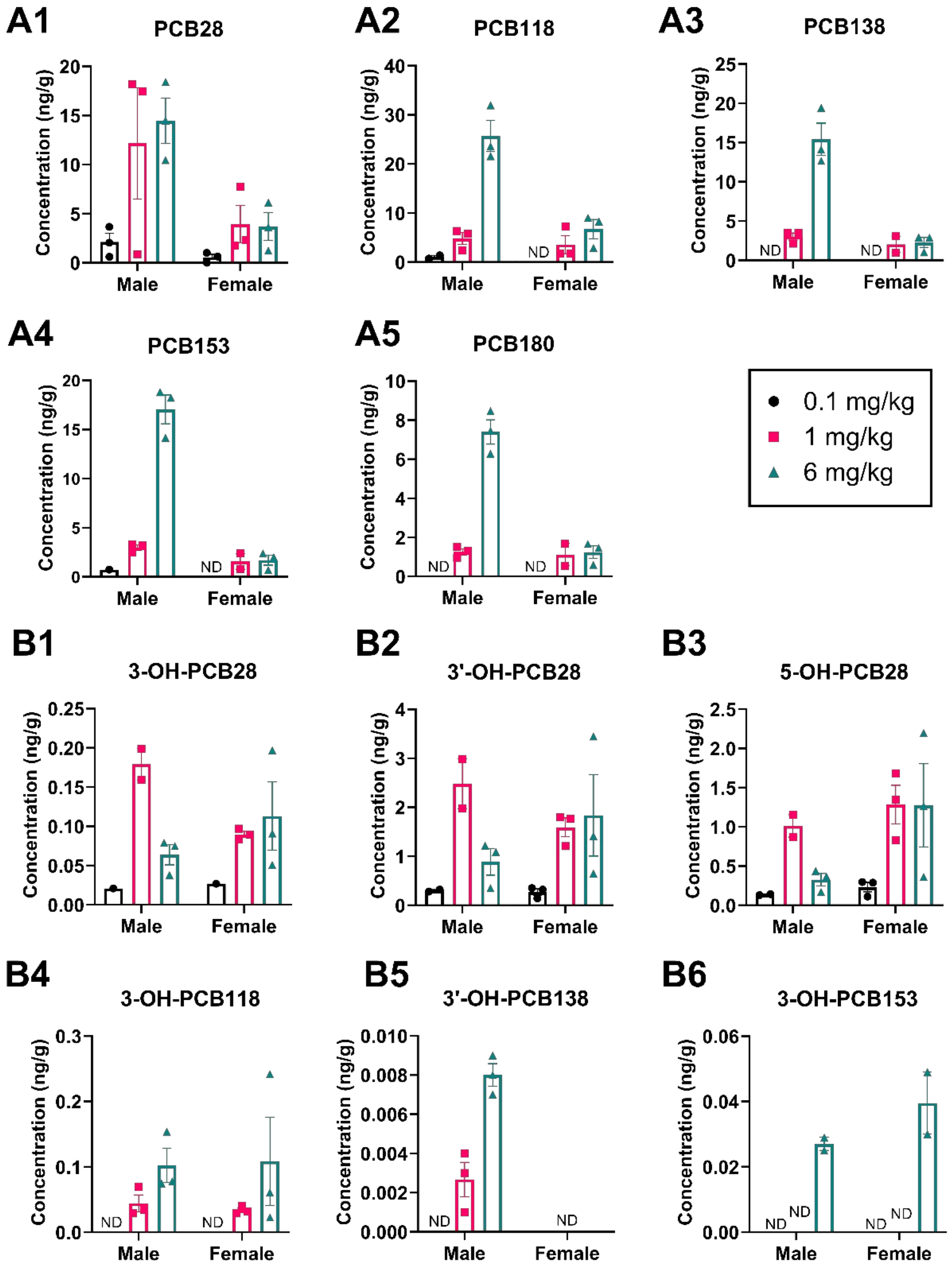

Figure S1. Weight adjusted concentrations (ng/g) of PCBs (A1-A5) and OH-PCBs (B1-B6) detected in pooled offspring urine. Data were shown as mean ± standard error (n = 3 pools/group). Values below LOD are non-detectable (ND) in the figures.

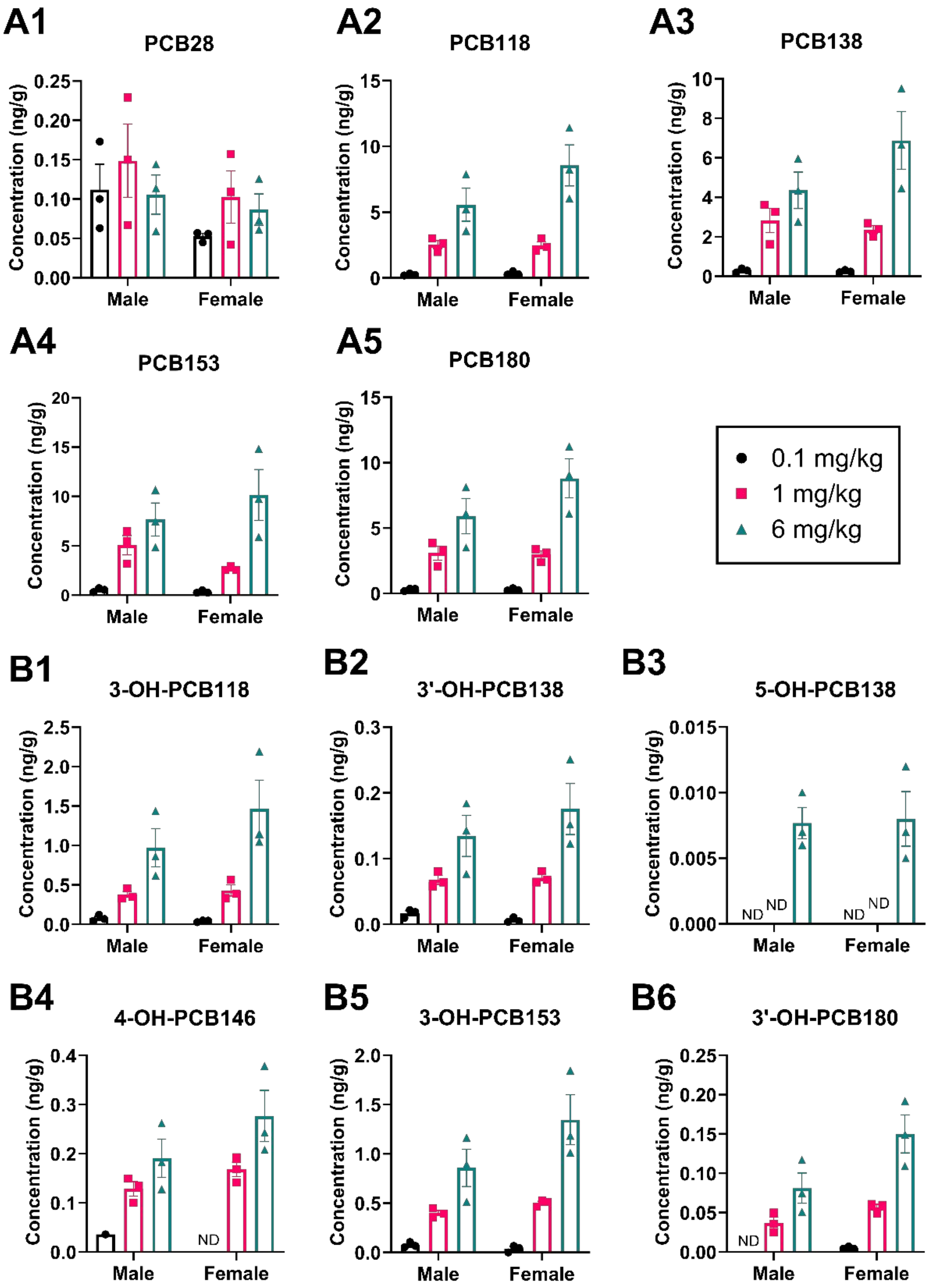

Figure S2. Weight adjusted concentrations (ng/g) of PCBs (A1-A5) and OH-PCBs (B1-B6) detected in pooled offspring whole blood. Data were shown as mean ± standard error (n = 3 pools/group). Values below LOD are non-detectable (ND) in the figures.

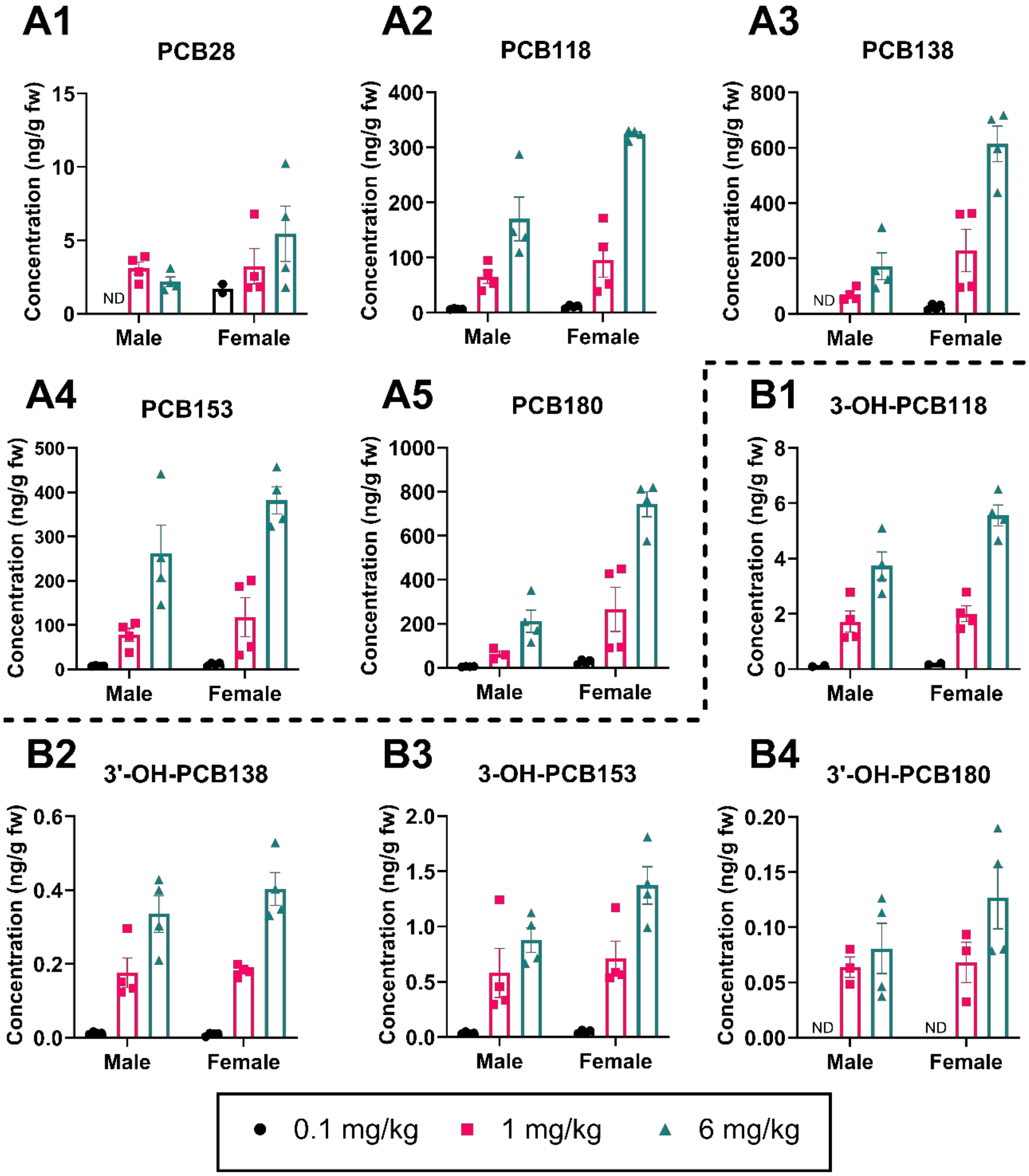

Figure S3. Weight adjusted concentrations (ng/g) of PCBs (A1-A5) and OH-PCBs (B1-B4) detected in offspring liver. Data were shown as mean ± standard error (n = 4). Values below LOD are non-detectable (ND) in the figures.

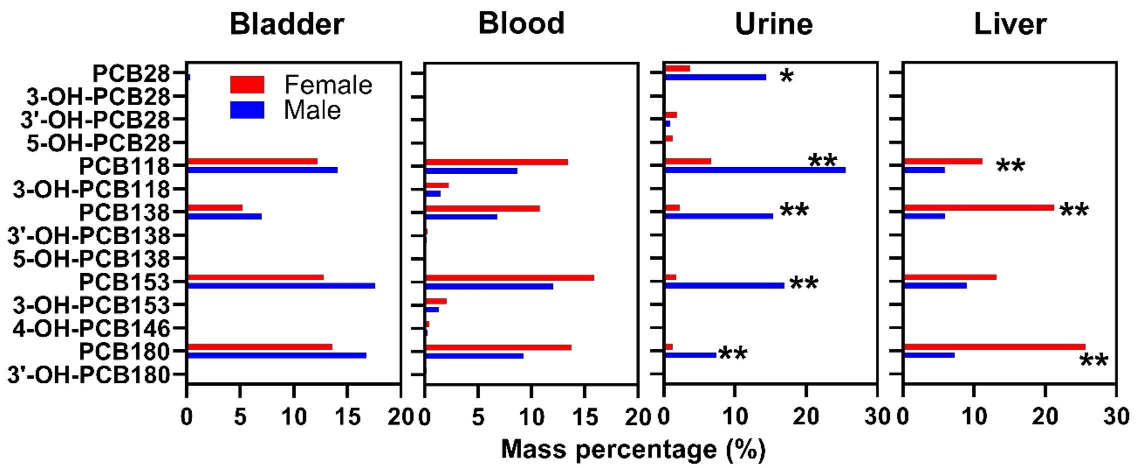

Figure S4. Mass percentage (%) of PCB and OH-PCB detected in high dose (6 mg/kg) PCB-exposed offspring bladder, blood, urine, and liver samples. The mass percentage is calculated using the formula = mass/(∑male+∑female). *p < 0.05, **p < 0.01 by Student's t-test.

References

(1) Black, T. H. The preparation and reactions of diazomethane. *Aldrichim Acta* **1983**, *16* (1), 3-10.

(2) Joshi, S. N.; Vyas, S. M.; Duffel, M. W.; Parkin, S.; Lehmler, H.-J. Synthesis of sterically hindered polychlorinated biphenyl derivatives. *Synthesis* **2011**, (7), 1045-1054. DOI:https://doi.org/10.1055/s-0030-1258454.

(3) Alam, S.; Carter, G.; Krager, K.; Li, X.; Lehmler, H.-J.; Aykin-Burns, N. PCB11 metabolite, 3,3'-dichlorobiphenyl-4-ol, exposure alters the expression of genes governing fatty acid metabolism in the absence of functional sirtuin 3: examining the contribution of MnSOD. *Antioxidants* **2018**, *7* (9), E121. DOI:https://doi.org/10.3390/antiox7090121.

(4) Rodriguez, E. A.; Li, X.; Lehmler, H. J.; Robertson, L. W.; Duffel, M. W. Sulfation of lower chlorinated polychlorinated biphenyls increases their affinity for the major drug-binding sites of human serum albumin. *Environ Sci Technol* **2016**, *50* (10), 5320-5327. DOI:https://doi.org/10.1021/acs.est.6b00484.

(5) Li, X.; Parkin, S.; Duffel, M. W.; Robertson, L. W.; Lehmler, H.-J. An efficient approach to sulfate metabolites of polychlorinated biphenyls. *Environ Int* **2010**, *36*, 843-848. DOI:https://doi.org/10.1016/j.envint.2009.02.005.

(6) Lehmler, H. J.; Robertson, L. W. Synthesis of hydroxylated PCB metabolites with the Suzuki-coupling. *Chemosphere* **2001**, *45* (8), 1119-1127.

(7) Lehmler, H. J.; Robertson, L. W. Synthesis of polychlorinated biphenyls (PCBs) using the Suzuki-coupling. *Chemosphere* **2001**, *45* (2), 137-143.

(8) Saktrakulkla, P.; Dhakal, R. C.; Lehmler, H. J.; Hornbuckle, K. C. A semi-target analytical method for quantification of OH-PCBs in environmental samples. *Environ Sci Pollut Res* **2020**, *27* (9), 8859-8871. DOI:https://doi.org/10.1007/s11356-019-05775-x.

(9) Dhakal, R.; Li, X.; Parkin, S. R.; Lehmler, H.-J. Synthesis of mono-and dimethoxylated polychlorinated biphenyls derivatives starting from fluoroarene derivatives. *Environ Sci Pollut Res* **2020**, *27*, 8905-8925. DOI:https://doi.org/10.1007/s11356-019-07133-3.

(10) Zhai, G. S.; Lehmler, H. J.; Schnoor, J. L. New hydroxylated metabolites of 4-monochlorobiphenyl in whole poplar plants. *Chem Cent J* **2011**, *5*. DOI:https://doi.org/10.1186/1752-153X-5-87.

(11) Li, X.; Holland, E. B.; Feng, W.; Zheng, J.; Dong, Y.; Pessah, I. N.; Duffel, M. W.; Robertson, L. W.; Lehmler, H.-J. Authentication of synthetic environmental contaminants and their (bio)transformation products in toxicology: polychlorinated biphenyls as an example. *Environ Sci Pollut Res* **2018**, *25* (17), 16508-16521. DOI:https://doi.org/10.1007/s11356-017-1162-0.

(12) McLean, M. R.; Bauer, U.; Amaro, A. R.; Robertson, L. W. Identification of catechol and hydroquinone metabolites of 4-monochlorobiphenyl. *Chem Res Toxicol* **1996**, *9* (1), 158-164. DOI:https://doi.org/10.1021/tx950083a.

(13) Kania-Korwel, I.; Zhao, H.; Norstrom, K.; Li, X.; Hornbuckle, K. C.; Lehmler, H.-J. Simultaneous extraction and clean-up of PCBs and their metabolites from small tissue samples

using pressurized liquid extraction. *J Chromatogr A* **2008**, *1214*, 37-46. DOI:https://doi.org/10.1016/j.chroma.2008.10.089.

(14) Dhakal, K.; He, X.; Lehmler, H. J.; Teesch, L. M.; Duffel, M. W.; Robertson, L. W. Identification of sulfated metabolites of 4-chlorobiphenyl (PCB3) in the serum and urine of male rats. *Chem Res Toxicol* **2012**, *25* (12), 2796-2804. DOI:https://doi.org/10.1021/tx300416v.

(15) Wang, H.; Bullert, A. J.; Li, X.; Stevens, H.; Klingelhutz, A. J.; Ankrum, J. A.; Adamcakova-Dodd, A.; Thorne, P. S.; Lehmler, H. J. Use of a polymeric implant system to assess the neurotoxicity of subacute exposure to 2,2',5,5'-tetrachlorobiphenyl-4-ol, a human metabolite of PCB 52, in male adolescent rats. *Toxicology* **2023**, *500*, 153677. DOI:https://doi.org/10.1016/j.tox.2023.153677.

(16) Marek, R. F.; Thorne, P. S.; Wang, K.; Dewall, J.; Hornbuckle, K. C. PCBs and OH-PCBs in serum from children and mothers in urban and rural U.S. communities. *Environ Sci Technol* **2013**, *47* (7), 3353-3361. DOI:https://doi.org/10.1021/es304455k.
